# High-dose chemotherapy as initial salvage chemotherapy in patients with relapsed or refractory testicular cancer: a systematic review and meta-analysis

**DOI:** 10.3389/fonc.2024.1437574

**Published:** 2024-10-01

**Authors:** Juan Briones, Pamela Diaz, Brian D. Nicholson

**Affiliations:** ^1^ Department for Continuing Education, University of Oxford, Oxford, United Kingdom; ^2^ Department of Hematology and Oncology, Pontificia Universidad Católica de Chile, Santiago, Chile; ^3^ Department of Clinical Immunology and Rheumatology, Pontificia Universidad Católica de Chile, Santiago, Chile; ^4^ Nuffield Department of Primary Care Health Sciences, University of Oxford, Oxford, United Kingdom

**Keywords:** refractory germ cell tumors, relapsed germ cell tumor, high dose chemotherapy with autologous stem cell transplantation, chemotherapy, survival, adverse (side) effects

## Abstract

**Background:**

The role of high-dose chemotherapy followed by autologous hematopoietic cell transplantation in the management of patients with relapsed/refractory germ-cell tumors has not been established in prospective studies. Our aim was to estimate the benefits and harm of this treatment in men with relapsed/refractory germ-cell tumors.

**Methods:**

Electronic databases, conference proceedings, and trial registers until April 30, 2023, were searched. Randomized and non-randomized prospective controlled trials were included. Risk of bias assessments were performed using either RoB2 or ROBINS-I tools. The certainty of evidence was assessed using the Grading of Recommendations, Assessment, Development, and Evaluations (GRADE) approach. Time-to-event data were analyzed using the hazard ratio. The primary outcome was overall survival, and a meta-analysis was not conducted to assess it because non-randomized trials were judged to have a critical risk of bias. Categorical data were analyzed using a risk ratio. All results are presented with the corresponding 95% confidence interval.

**Results:**

Four out of 3,824 records met the inclusion criteria, and three out of four were used to assess primary and secondary outcomes. Based on the IT94 study (N = 263 participants), single high-dose chemotherapy followed by autologous hematopoietic cell transplantation may have little to no effect on overall survival [hazard ratio (HR) 0.98, 95%CI 0.68 to 1.42; p = 0.916]. Non-randomized trials (N = 43 participants) showed contrasting results, which may be explained by the number of cycles of high-dose chemotherapy administered in each study. Regarding secondary outcomes, information was only provided for event-free survival, response rate, and acute toxicities.

**Conclusions:**

Based on prospective data, there is insufficient evidence to support or refute the proposal that high-dose chemotherapy with autologous hematopoietic cell transplantation improves survival in men with relapsed/refractory germ-cell tumors. If this treatment is considered essential, the choice should be made by experienced clinicians at high-volume cancer centers.

## Background

The role of high-dose chemotherapy (HDCT) followed by autologous hematopoietic cell transplantation (AHCT) in the management of patients with relapsed or refractory germ-cell tumors (GCTs) has not been established in prospective studies. This intervention is expensive and may be associated with severe hematological and non-hematological toxicities, as well as death, while the impact of HDCT on quality of life (QoL) is largely unknown.

Testicular GCT is a highly curable malignancy. In fact, roughly 90% of all testicular GCTs may be cured with medical treatment (1, 2). The majority of stage I testicular GCT patients are cured after radical orchiectomy. Stage II and III (metastatic) GCT patients undergo further treatments after surgery, including platinum-based chemotherapy, radiotherapy, or retroperitoneal lymph node dissection.

Experience matters in GCT management. Analyses from the Swedish Norwegian Testicular Cancer Project and Scotland showed that patients with metastatic GCTs had better survival rates when they were treated at high-volume centers compared with those treated at low-volume hospitals (3).

According to the The International Germ Cell Cancer Collaborative Group (IGCCCG) Update Consortium, progression-free survival (PFS) and overall survival (OS) for metastatic seminoma and non-seminoma significantly improved between 1990 and 2013 (1, 2). Despite these improvements, the 3-year overall survival of metastatic patients with relapsed or refractory disease after first-line platinum-based chemotherapy ranges from 6.1% to 77% (4). Regrettably, it is a matter of debate which is the best course of action across prognostic categories. The most relevant clinical practice guidelines in the management of germ-cell malignancies do not show a clear recommendation regarding the treatment of relapsed/refractory GCTs after first-line chemotherapy (5, 6). Guidelines and experts do provide a clear statement in two rare clinical scenarios: “growing teratoma” syndrome during or after platinum-based chemotherapy and resectable late relapsed GCTs, where salvage surgery would be the best treatment option (7, 8). In situations where chemotherapy is the cornerstone salvage therapy, it is still controversial if HDCT has a superior effect to conventional-dose chemotherapy (CDCT). In fact, several studies with contradictory results have been published on this topic, including phase I, II, and III clinical trials and retrospective studies as well as systematic reviews.

In theory, increasing the dose of chemotherapy may amplify the treatment efficacy. High doses of chemotherapy may overcome the resistance of germ-cell tumor cells to conventional-dose regimens and improve survival. However, HDCT produces dose-limiting toxicities, making the bone marrow the most affected tissue that can be salvaged using stem cell transplant (9).

The ability to safely deliver HDCT followed by AHCT is strongly associated with the medical team’s experience. Actually, it is well known that death rates due to toxicity are largely related to expertise (10, 11). Patients receiving HDCT require several supportive treatments and teamwork. Supportive care needed during treatment includes AHCT, granulocyte-colony stimulating factor, transfusion of blood products, and intensive care unit support.

Many unknowns exist regarding HDCT regimens, including the number of cycles, drugs, and the need for induction chemotherapy. This reflects what happens in daily clinical practice worldwide (12).

Two phase III randomized controlled trials (RCTs) have tested the efficacy of HDCT in previously treated GCT patients (13, 14). In addition, a current international phase III RCT is assessing the efficacy of Paclitaxel, Ifosfamide and Cisplatin (TIP) given at first relapse compared with Paclitaxel and Ifosfamide followed by Carboplatin and Etoposide (TI-CE) followed by AHCT (15). The IT94 trial used four cycles of Etoposide, Ifosfamide and Cisplatin (VIP)/Vinblastin, Ifosfamide and Cisplatin (VeIP) compared with three cycles of VIP/VeIP followed by one cycle of HDCT with AHCT. The second phase III trial compared one cycle of VIP followed by three cycles of high-dose chemotherapy with carboplatin and etoposide (arm A) versus three cycles of VIP and one cycle of HDCT while adding cyclophosphamide to carboplatin and etoposide (arm B). This study was terminated prematurely due to excess deaths in arm B. None of these studies produced meaningful results in terms of OS.

The most meaningful data regarding HDCT come from retrospective studies. A study conducted at Indiana University treated 364 patients with either one or two courses of carboplatin and etoposide followed by AHCT between 2004 and 2014. The 2-year PFS was 60%, and the 2-year OS was 66% at a median follow-up of 3.3 years (16). Furthermore, Feldman et al. (17) reported a full dataset of 107 patients treated with the TI-CE regimen. This study showed a 5-year disease-free survival (DFS) and OS of 47% and 52% (median follow-up, 61 months), respectively.

Finally, systematic reviews (SRs) already published have based their conclusions mainly on survival endpoints (e.g., OS), forgetting other patient-important outcomes, including adverse events and QoL. Furthermore, those reviews have some methodological limitations, namely, no available protocol, no adherence to the Preferred Reporting Items for Systematic Reviews and Meta-Analyses (PRISMA) guidelines, no attempt to perform risk of bias assessment in included studies, and did not assess the certainty of the evidence (18, 19).

This SR and meta-analysis aimed to assess the efficacy of HDCT followed by AHCT versus CDCT in improving OS, event-free survival (EFS), response rate, and PFS, determine the treatments’ impact on QoL and describe the treatment-related toxicities in men with relapsed or refractory GCTs.

## Methods

This SR was developed with the guidance of the PRISMA 2020 statement (20, 21). The full protocol is available in the **Supplementary Material**.

### Research question

Does high-dose chemotherapy followed by autologous hematopoietic cell transplantation as initial salvage chemotherapy in patients with relapsed or refractory germ-cell tumors improve outcomes compared with conventional-dose chemotherapy?

*Primary outcome*: overall survival.

*Secondary outcomes*: quality of life, event-free survival, response rate, progression-free survival, and acute and chronic toxicities.

### Eligibility criteria

#### Studies

Randomized and non-randomized prospective controlled trials comparing the effectiveness of HDCT followed by AHCT with CDCT for people with relapsed/refractory GCTs were included.

#### Participants

Participants were men (≥15 years old at the date of diagnosis) with a diagnosis of GCTs, confirmed by either pathology or elevated tumor markers plus suggestive imaging, and patients with unequivocal evidence of relapse or progression after first-line platinum-based chemotherapy for metastatic GCTs. Patients with extragonadal GCTs [retroperitoneum, mediastinum, or central nervous system (CNS)] were included if they met the aforementioned criteria.

#### Interventions

We used the intervention HDCT followed by AHCT (i.e., experimental arm) and CDCT (i.e., control arm). There are various HDCT and CDCT regimens; however, most of them share at least two chemotherapy drugs. Most HDCT regimens incorporate etoposide and carboplatin as cornerstone drugs. In contrast, the majority of CDCT regimens also include two keystone chemotherapies: ifosfamide and cisplatin. Hence, we considered all of them as one experimental arm (HDCT) and one control arm (CDCT).

We also decided to include in the experimental arm the HDCT regimens that incorporated induction chemotherapy as well as HDCT regimens given in either a single or sequential way.

### Search methods

#### Electronic databases

MEDLINE PubMed, Embase, and CENTRAL electronic databases were searched from inception to April 30, 2023 (see searching strategy in **Appendix 1–3** in the **Supplementary Material**). Language restrictions were not imposed.

#### Searching other resources

Reference lists of selected review articles were searched (9, 19, 22). Conference proceedings of the American Society of Clinical Oncology (ASCO), the European Society for Medical Oncology (ESMO), the International Society for Paediatric Oncology, the American Society of Pediatric Hematology/Oncology, the American Society for Blood and Marrow Transplantation, and the European Society for Blood and Marrow Transplantation were also searched from inception to April 30, 2023. ClinicalTrials.gov and the WHO International Clinical Trials Registry Platform were scanned for ongoing trials (from inception to April 30, 2023).

### Data collection and analysis

#### Selection of studies

**Figure 1:**
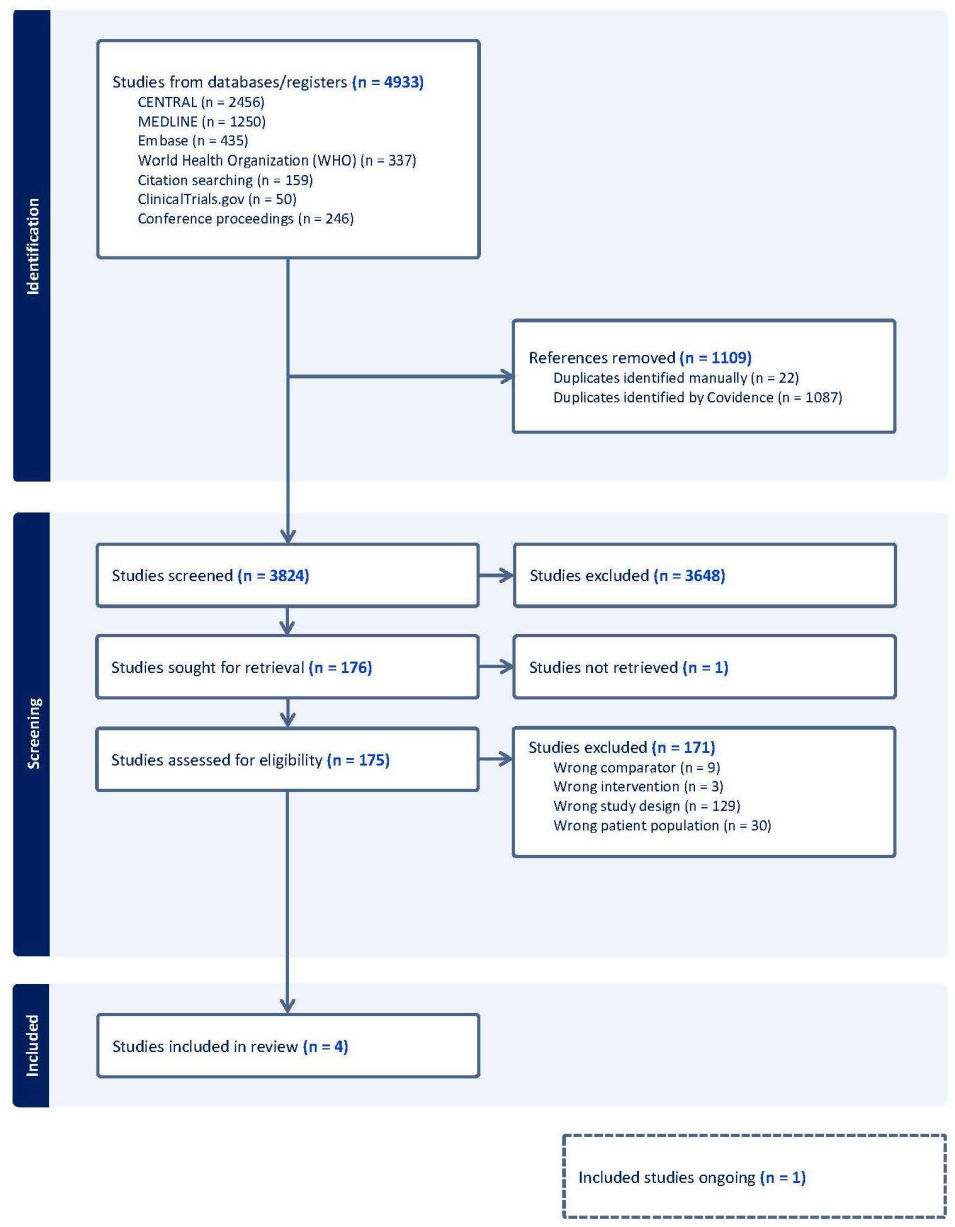
PRISMA flow diagram. PRISMA, Preferred Reporting Items for Systematic Reviews and Meta-Analyses.

We uploaded all titles and abstracts retrieved by electronic searching into Covidence and removed any duplicates. One reviewer (JB) examined the remaining references and excluded any studies that did not meet the inclusion criteria. Then, we collected the full text of the studies that met the inclusion criteria based on the title, abstract, or both for detailed inspection. Two reviewers (JB and PD) independently assessed the eligibility of the retrieved papers and resolved any discrepancies through discussion. A PRISMA flow diagram was produced (**Figure 1**).

#### Data extraction and management

Two authors (JB and PD) independently extracted data using a customized template. Discrepancies were resolved between reviewers by discussion. Data on the characteristics of the study, participants, interventions, methods, outcomes, and details of funding sources for the included studies were extracted. Hazard ratio (HR) calculations spreadsheet was used to facilitate the estimation of HRs from included studies (23).

We extracted data to enable intention-to-treat (ITT) and per-protocol analyses.

#### Risk of bias assessment

Two authors (JB and PD) independently assessed the risk of bias in the included studies using either RoB2 (24) or ROBINS-I (25) tools according to the study design. Discrepancies were resolved between reviewers by discussion. The risk of bias was separately assessed for each outcome.

#### Measures of treatment effect

Time-to-event data were analyzed using HR, and categorical data were analyzed using Response rate (RR). All results are presented with the corresponding 95% confidence interval. A p-value ≤ 0.05 was considered statistically significant.

The outcomes of the included studies did not have homogeneous definitions. Therefore, we decided to accept the definitions provided by the authors of the original studies.

#### Missing data

ITT analysis was applied to all outcomes when reporting results. A per-protocol analysis was utilized to assess adverse events (AEs). We would attempt to contact the study authors of the included studies.

#### Data synthesis

Meta-analysis for OS was not conducted because only one study had reliable data for analysis. The generic inverse–variance method was used to calculate time-to-event outcomes, considering that we could obtain log HR and standard errors from included studies.

For categorical data, we decided to conduct meta-analyses using the Mantel–Haenszel method since it is more robust for sparse data (26).

We employed STATA 17.0 to perform statistical analysis (see STATA commands in the **Supplementary Material**).

#### Heterogeneity

Variability would have been investigated and handled following the Ann Arbor Heterogeneity Consensus Group recommendations (27, 28) if we had been capable of conducting a meta-analysis of OS (see the protocol in the **Supplementary Material** for further details).

-**Prespecified clinical covariates.**
We planned to investigate five variables with strong rationale: histological subtype, baseline risk as per International Prognostic Factors Study Group (IPFSG) prognostic score, type of CDCT regimen, type of HDCT regimen, and number of cycles of HDCT.
Regrettably, we were not able to conduct subgroup analysis and meta-regression as we stated in the protocol.
-**Role of statistical heterogeneity**. A non-significant Cochran’s Q test (p-value ≥ 0.1) or a small I^2^ (<25%) would not have avoided the need to investigate clinical and methodological heterogeneity if we had been able to perform a meta-analysis of OS.
We did conduct meta-analyses to explore some secondary outcomes further. However, due to the exploratory nature of secondary outcomes, clinical and methodological heterogeneities were not investigated.
-**Plotting and visual aids**. We used a graphical presentation of the data included from studies (e.g., forest plots). A funnel plot was not used due to few available studies.

#### Dealing with multiplicity

We followed the next strategies for dealing with each source of multiplicity as we explained in detail in the protocol:

-**Multiple outcomes**. We classified the outcomes into primary and secondary outcomes. Secondary outcomes were not part of the main conclusion of the review.
-**Multiple groups.** There are several HDCT and CDCT regimens; however, most of them share at least two chemotherapy drugs. Thus, we considered all of them as one intervention group and one control group.
-**Multiple time points**. We combined information at different time points by assuming that the HR (as a summary estimate effect) is constant over time (29).
-**Multiple effect measures**. OS and EFS can be measured using different metrics (e.g., median, 5-year rate, or HR). Since sufficient data were available, we calculated the HR for each time-to-event outcome.
-**Subgroup analyses.** Subgroup analyses were not conducted.
-**Multiple sources.** We maximized the information yield by collating all available data (duplicate publications, companion documents, or multiple reports of a primary trial). We used the most complete dataset aggregated across all known publications.

### Summary of findings and assessment of the certainty of the evidence

We prepared a summary of the findings table using GRADEpro (30). For each outcome, a reviewer (JB) assessed the certainty of the evidence using the five GRADE (Grading of Recommendations, Assessment, Development, and Evaluations) considerations (31).

## Results

### Description of the studies

#### Results of the search

Four trials fulfilled the inclusion criteria (**Figure 1**) (13, 15, 32, 33). One of them is not published yet (15). Therefore, three out of four were used to assess primary and secondary outcomes. The characteristics of the included studies are summarized in **Tables 1A–D**. See the characteristics of the excluded studies in the **Supplementary Material** (**Supplementary Table S1**).

**Table 1:** Characteristics of the included studies.

Table 1A	
Mardiak (2000) (32)	
Study characteristics	
**Methods**	Non-randomized prospective controlled study. Single center. Country: Slovakia. Median follow-up: 37 months.
**Participants**	Number of participants: 25 participants (intervention/control = 11/14 patients). 1.6 VIP was administered at the beginning of the study, and later, on the basis of good tolerance, the last 11 patients received 1.9 VIP with AHCT. Median age: 33 years (range 18–55 years). Number of lines of chemotherapy: median 2 lines. Previous use of platinum-based chemotherapy: 16 patients. The rest of participants were treatment-naïve (n = 9). Inclusion criteria: - Testicular cancer patients with poor prognosis based on IGCCCG categories. - Relapsed/refractory testicular cancer after platinum-based chemotherapy. Exclusion criteria: - No patient was excluded from this trial on the basis of metastatic site, performance status, poor response or death during the initial course of chemotherapy, or platinum resistance in previous chemotherapy regimens.
**Intervention**	Participants received 1 cycle of 1.6 VIP followed by 3 cycles of HDCT with AHCT. - 1.6 VIP. Etoposide 600 mg/m^2^, ifosfamide 8,000 mg/m^2^, and cisplatin 100 mg/m^2^. - 1.9 VIP. Etoposide 712.5 mg/m^2^, ifosfamide 9,500 mg/m^2^, and cisplatin 100 mg/m^2^. In the 1.9 VIP regimen group, the first cycle was administered at 1.6 relative dose intensity level followed by PBSC^a^ collection, and in subsequent 3 courses, patients received 1.9 VIP chemotherapy. In each of these 3 courses of chemotherapy, the PBSCs were reinfused on day 8 and followed by administration of G-CSF^b^ or GM-CSF^c^.
**Comparator**	Participants received 4 cycles of 1.6 VIP. - 1.6 VIP. Etoposide 600 mg/m^2^, ifosfamide 8,000 mg/m^2^, and cisplatin 100 mg/m^2^. After each course of 1.6 VIP chemotherapy, patients received GM-CSF or G-CSF in a dose 5 μg/kg per day for 13 days.
**Outcomes**	Response rate - CR was defined as the complete disappearance of all objective evidence of disease including the decrease of HCG and/or AFP to the normal limits for at least 1-month duration. - The decrease of HCG and/or AFP to the normal limit but with persistence of tumor masses on the CT was classified as PRm−. - Patients who achieved other results than CR or PRm− were classified as treatment failures. Overall survival at 1 year and 2 years. Toxicity grade 3–5^d^.
**Notes**	No data were provided to estimate IPFSG prognostic score. QoL, PFS, and chronic toxicities were not evaluated in this study. There was no detail information about the primary site and sites of metastasis. Lack of granularity about time-to-event outcomes and baseline characteristic of the included patients. Data from Mardiak's study were not part of the main conclusion of the Systematic review.

^a^PBSC, peripheral blood stem cells.

^b^G-CSF, granulocyte colony-stimulating factor.

^c^GM-CSF, granulocyte-macrophage colony-stimulating factor.

^d^Based on Common Terminology Criteria for Adverse Events (CTCAE).

**Table 1B:** 

Pico (2005) (13)^a^	
Study characteristics	
**Methods**	Randomized prospective controlled trial. Multicenter (tertiary centers in Europe). Country: France. Median follow-up: 45 months (range 22 days to 7.5 years).
**Participants**	Number of participants: 280 participants. Seventeen patients were considered ineligible after randomization. 135 and 128 participants were assigned to HDCT with AHCT and CDCT, respectively. Median age. HDCT arm: 29 years (range 16–55 years), and CDCT arm: 30 years (range 15–58 years). Primary site: approximately 83% had testicular GCT. Histology subtype: roughly 9% had seminoma. Number of lines of chemotherapy: 1. Previous use of platinum-based chemotherapy: yes (85% of patients received BEP or EP as first-line treatment). Inclusion criteria: - Male patients at least 16 years old with performance status 0–2. - Relapsing GCT patients who had achieved a complete or partial remission from platinum combination chemotherapy as first-line treatment. Patients with elevated tumor markers, metastases, or seminoma failing cisplatin combination chemotherapy were also included. Exclusion criteria: - Refractoriness to first-line platinum-containing chemotherapy, defined as a documented increase of tumor burden and/or serum tumor marker level within 1 month of treatment. - Pure seminoma pretreated with carboplatin.
**Intervention**	Participants received 3 cycles of VIP/VeIP followed by 1 cycle of HDCT with AHCT. - Both CDCT regimens included ifosfamide (1,200 mg/m^2^, i.v.), mesna (400 mg/m^2^, i.v.), and cisplatin (20 mg/m^2^, i.v.), days 1–5. Each cycle included either etoposide (75 mg/m^2^, i.v., days 1–5) (PEI) or vinblastine (0.11 mg/kg, days 1–2) (VeIP), depending on which drug had previously been used as first-line treatment. - CarboPEC regimen included a 1- or 2-h carboplatin infusion day 1, etoposide (450 mg·m^−2^·day^−1^, i.v.), cyclophosphamide (1,600 mg·m^−2^·day^−1^, i.v.), and mesna (3,600 mg·m^−2^·day^−1^; 1-h infusion) on days 1–4, followed on day 7 by either bone marrow or stem cell autologous hematopoietic re-infusion. Carboplatin dosages of 0, 250, 400, and 550 mg·m^−2^·day^−1^ were determined on the basis of EDTA clearance: <30, <60, <100, and ≥100 mL/min, respectively.
**Comparator**	Participants received 4 cycles of VIP/VeIP. - Both regimens included ifosfamide (1,200 mg/m^2^, i.v.), mesna (400 mg/m^2^, i.v.), and cisplatin (20 mg/m^2^, i.v.), days 1–5. Each cycle included either etoposide (75 mg/m^2^, i.v., days 1–5) (PEI) or vinblastine (0.11 mg/kg, days 1–2) (VeIP), depending on which drug had previously been used as first-line treatment.
**Outcomes**	Event-free survival was calculated from the date of start of the salvage chemotherapy cycle to disease progression, relapse, or death due to any cause. 26% of patients were randomized after the start of the first cycle. Disease-free survival was calculated from the date of start of the salvage chemotherapy cycle to failure after achieving a CR. Overall survival was calculated from the date of start of the salvage chemotherapy cycle to death due to any cause. Response rate: - cCR: complete disappearance by all lesions and serum TMs normalization for at least 1 month from chemotherapy alone. - pCR: normal TMs, complete resection of non-viable malignancies (necrosis, fibrosis, and teratoma). - sCR: no evidence of disease after complete resection of viable malignancy and normal TMs. - PRm+: partial remission with elevated TMs. - PRm− partial remission or >90% reduction of elevated TMs for at least 1 month. - SD: response not qualifying as either PR or PD. - PD: PD from chemotherapy or >10% increase in elevated TMs. Acute toxicity grade 3–5.
**Notes**	No data were provided to estimate IPFSG prognostic score. QoL, PFS, and toxicities were not evaluated in this study. EFS data could not be extracted for each intervention group. Only 5 patients met the inclusion criteria in this cohort. Lack of information about doses of CDCT and HDCT. Data from this study were not used to build the main conclusions of the systematic review.

^a^ Dr José Luis Pico (1940–2022) left us on July 30, 2022, surrounded by all his loved ones.

**Table 1C:** 

Faure-Conter (2014) (33)	
Study characteristics	
**Methods**	Non-randomized prospective controlled trial. Multicenter (tertiary centers in France). Country: France. Range follow-up: 3.6 years to 9.5 years (length to follow-up was not reported in some patients).
**Participants**	Number of participants: 19 participants (male and female). Patients were treated according to their baseline prognosis. Median age. HDCT arm: 7.25 years (range: 12 months to 19 years), and CDCT arm: 10 years (range: 12 months to 17 years). Primary site: approximately 37% had testicular or mediastinal/retroperitoneal GCT. Histology subtype: 100% had non-seminoma. Number of lines of chemotherapy: 1. Previous use of platinum-based chemotherapy: yes. Inclusion criteria: - In case of non-remission (i.e., non-normalization of TM and incomplete resection of viable tumor), progression during chemotherapy, or recurrent malignant NSGCT, salvage chemotherapy was recommended. - The surgical removal of all persistent residues was recommended before or after salvage chemotherapy. - Radiotherapy was not recommended as initial or salvage treatment. - Only patients initially treated with chemotherapy who relapsed or progressed were included. Exclusion criteria: - Non-malignant relapses (e.g., pure mature teratoma) or non-GCT malignancies (e.g., leukemia) occurring after chemotherapy were excluded.
**Intervention**	High-risk^a^ participants received HDCT. - Etoposide and thiotepa (5 patients), carboplatin, etoposide, and cyclophosphamide (4 patients), and unknown HDCT regimen (1 patient).
**Comparator**	Intermediate-risk^b^ patients received CDCT. - Platinum salts-based regimens or various other combination therapies based on taxotere, oxaliplatin, gemcitabine (TOG) or vinorelbine, ifosfamide, and farmorubicine (NIF).
**Outcomes**	Event-free survival was calculated as the date from the first relapse to a new documented progression or death due to any cause. Overall survival was defined as the time from the first relapse to the death due to any cause. Response rate: - CR was defined as the clinical, radiographical, and biochemical disappearance of all disease features. - PD was defined as an increase in the tumor volume, the occurrence of new metastases, or increased TM levels. - Incomplete remission (IR) was defined as any response observed lower than a CR.
**Notes**	No data were provided to estimate IPFSG prognostic score. QoL, PFS, and toxicities were not evaluated in this study. EFS data could not be extracted for each intervention group. Only 5 patients met the inclusion criteria in this cohort. Lack of information about doses of CDCT and HDCT. Data from this study were not used to build the main conclusions of the systematic review.

^a^ High-risk GCT: partially removed tumor either metastatic or AFP ≥ 15,000 ng/mL.

^b^ Intermediate-risk GCT: partially removed non-metastatic tumors and AFP < 15,000 ng/mL.

**Table 1D:** 

Feldman (2018) (15)	
Study characteristics	
**Methods**	Randomized prospective controlled trial. Multicenter (tertiary centers in the USA, Europe, and Oceania). Country: USA. Estimated study completion date: June 2024.
**Participants**	Number of participants: 420 testicular cancer patients. Inclusion criteria: - Confirmation of GCT histology on pathological review at the center of enrollment. - Tumor may have originated in any primary site. - Pathological confirmation may not be required if a clinical situation is consistent with the diagnosis of GCT. - Evidence of progressive or recurrent GCT following one line of cisplatin-based chemotherapy. - Must have received 3–6 cycles of cisplatin-based chemotherapy as part of first-line chemotherapy. - Prior treatment with carboplatin as adjuvant therapy is allowed. - Prior treatment with 1–2 cycles of BEP or EP as adjuvant chemotherapy for early Stage GCT is allowed. - Age ≥ 14 years (≥18 years in Germany) and male. - ECOG Performance Status 0 to 2. Exclusion criteria: - No prior treatment with high-dose chemotherapy (defined as treatment utilizing stem cell rescue). - No prior treatment with TIP. - No concurrent treatment with other cytotoxic drugs or targeted therapies. - No radiation therapy (other than to the brain) within 14 days of day 1 of protocol chemotherapy. - No previous chemotherapy within 17 days prior to enrollment. - No late relapse with completely surgically resectable disease. - No secondary somatic malignancy arising from teratoma when it is actively part of the disease recurrence or progression.
**Intervention**	Participants received TI-CE followed by AHCT. - TI. Cycles 1–2 (1 cycle = 14 days). Paclitaxel 200 mg/m^2^ IV over 3 hours on Day 1, ifosfamide 2,000 mg/m^2^ IV daily on Days 1–3 with mesna protection, G-CSF 10 µg/kg subcutaneously on Days 3–15 (cycle 1) and Days 3–14 (cycle 2) or pegylated G-CSF 6 mg subcutaneous on Day 4 or 6 (cycle 1) and Day 4 or 5 (cycle 2). Leukapheresis every 14 days, if there is an inadequate number of CD34+ cells/kg collected in cycle 1. - CE. Cycles 3–5 (1 cycle = 21 days). Carboplatin daily on Days 1–3 and etoposide 400 mg/m^2^ daily on Days 1–3. Stem cell reinfusion on day 5. Pegylated G-CSF 6 mg subcutaneously or G-CSF at approximately 5 µg/kg daily on Days 5–15.
**Comparator**	Participants received TIP for 4 cycles. - TIP. Cycles 1–4 (1 cycle = 21 days). Paclitaxel 250 mg/m^2^ IV over 24 hours on Day 1, ifosfamide 1,500 mg/m^2^ IV daily on Days 2–5 with mesna protection, cisplatin 25 mg/m^2^ IV daily on Days 2–5, and pegylated G-CSF 6 mg subcutaneous on Day 6 or 7 or G-CSF as defined in the protocol on Days 6–18.
**Outcomes**	Primary outcome: - OS (time frame: up to 36 months post-treatment). Secondary outcomes: - PFS (time frame: up to 36 months post-treatment). - Proportion of patients achieving either a CR or PR (time frame: up to 3 months post-registration). - Treatment-related mortality (time frame: up to 30 days post-treatment). - Number of participants with treatment-related adverse events as assessed by CTCAE v4.0 (time frame: up to 3 months post-registration). - Validation of International Prognostic Factor Study Group stratification system (time frame: up to 3 years post-registration).
**Notes**	Not yet published. QoL was not among its primary and secondary outcomes

(A) Mardiak et al. (32). (B) Pico et al. (13). (C) Faure-Conter et al. (33). (D) Feldman et al. (2018).

### Risk of bias in included studies

#### Randomized controlled trials

**Figure 2:**
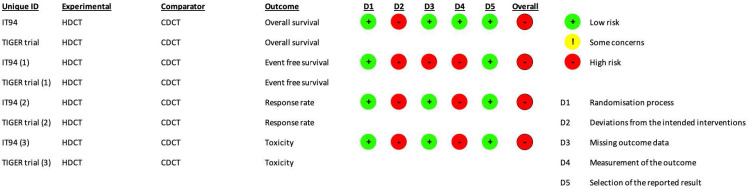
RoB2 risk of bias assessment of randomized controlled trials (intention-to-treat analysis). Risk of bias was separately assessed for each outcome.

**Figure 3:**
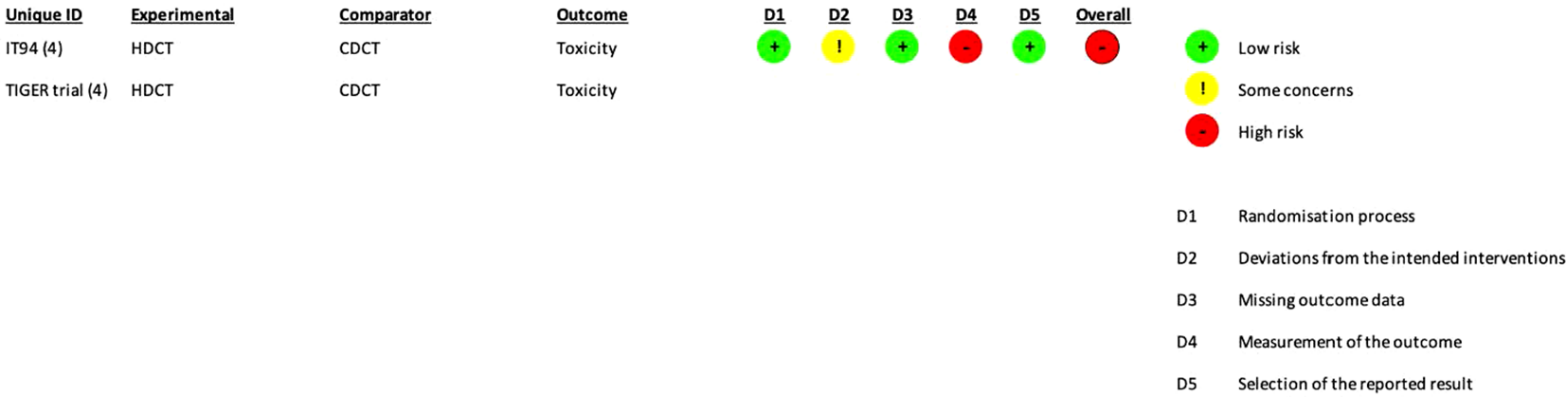
RoB2 risk of bias assessment of randomized controlled trials (per-protocol analysis). Risk of bias was only assessed for toxicity.

The risk of bias assessment and support judgments of the included RCTs (13) can be found in **Supplementary Tables S2A–E** and **Figures 2**, **3**.

The risk of bias was separately assessed for each outcome. For all outcomes, the aim was to assess the effect of assignment to intervention except toxicity (the effect of adhering to intervention was evaluated).

#### Non-randomized controlled trials

**Figure 4:**
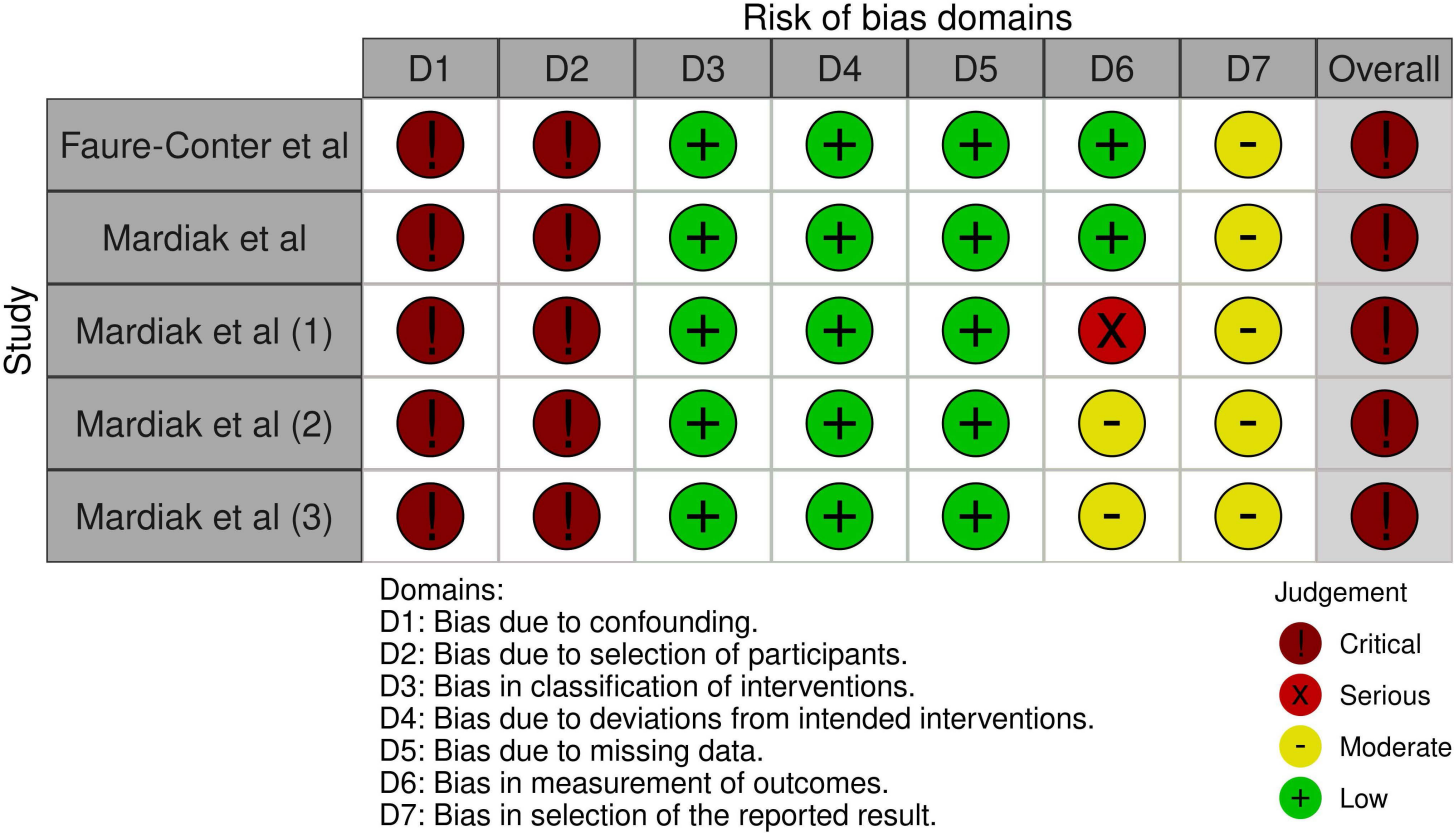
ROBINS-I risk of bias assessment of non-randomized controlled trials. Risk of bias was separately assessed for each outcome. Faure-Conter et al. (ITT analysis): overall survival. Mardiak et al. (ITT analysis): overall survival. Mardiak et al. (1) (ITT analysis): response rate. Mardiak et al. (2) (ITT analysis): toxicity. Mardiak et al. (3) (per-protocol analysis): toxicity. ITT, intention-to-treat.

The risk of bias assessment and support judgments of the included non-randomized prospective controlled trials (32, 33) can be found in **Supplementary Tables S3A–E** and **Figure 4** (34).

### Effects of interventions

See **Table 2**.

**Table 2:** Summary of findings.

High-dose chemotherapy as initial salvage chemotherapy in patients with relapsed or refractory testicular cancer: a systematic review and meta-analysis						
Patient or population: relapsed or refractory testicular cancer Setting: tertiary Intervention: high-dose chemotherapy followed by autologous hematopoietic cell transplantation Comparison: conventional-dose chemotherapy						
Outcomes	Anticipated absolute effects^*^ (95%CI)		Relative effect (95%CI)	No. of participants (studies)	Certainty of the evidence (GRADE)	Comments
	Risk with conventional-dose chemotherapy	Risk with high-dose chemotherapy followed by autologous hematopoietic cell transplantation				
Overall survival follow-up: median 45 months	**Low**		**HR 0.98** (0.68 to 1.42) [overall survival]	263 (1 RCT)	⊕○○○ Very low^a^	High-dose chemotherapy followed by autologous hematopoietic cell transplantation may reduce/have little to no effect on overall survival, but the evidence is very uncertain.
	445 per 1,000	**438 per 1,000** (330 to 567)				
Quality of life—not reported						No information was provided for QoL.
Event-free survival follow-up: median 45 months	**Low**		**HR 0.80** (0.59 to 1.10) [event-free survival]	263 (1 RCT)	⊕⊕○○ Low^b^	High-dose chemotherapy followed by autologous hematopoietic cell transplantation may improve event-free survival.
	648 per 1,000	**566 per 1,000** (460 to 683)				
Complete response	418 per 1,000	**422 per 1,000** (318 to 569)	**RR 1.01** (0.76 to 1.36)	247 (1 RCT)	⊕⊕○○ Low^c^	High-dose chemotherapy followed by autologous hematopoietic cell transplantation may result in little to no difference in complete response.
Partial response plus normal tumor markers	172 per 1,000	**184 per 1,000** (108 to 315)	**RR 1.07** (0.63 to 1.83)	247 (1 RCT)	⊕⊕○○ Low^c^	High-dose chemotherapy followed by autologous hematopoietic cell transplantation may result in little to no difference in partial response plus normal tumor markers.
Overall response rate	590 per 1,000	**608 per 1,000** (496 to 746)	**RR 1.030** (0.840 to 1.264)	247 (1 RCT)	⊕⊕○○ Low^c^	High-dose chemotherapy followed by autologous hematopoietic cell transplantation may result in little to no difference in overall response rate.
Failure	410 per 1,000	**392 per 1,000** (289 to 532)	**RR 0.956** (0.705 to 1.298)	247 (1 RCT)	⊕⊕○○ Low^c^	High-dose chemotherapy followed by autologous hematopoietic cell transplantation may not reduce failure.
Progression-free survival—not reported						No information was provided for PFS.
Neutropenia G3+	882 per 1,000	**935 per 1,000** (865 to 1,000)	**RR 1.06** (0.98 to 1.14)	274 (1 RCT)	⊕⊕⊕○ Moderate^d^	High-dose chemotherapy followed by autologous hematopoietic cell transplantation likely results in little to no difference in neutropenia G3+.
Febrile neutropenia G3+	493 per 1,000	**773 per 1,000**(640 to 941)	**RR 1.57**(1.30 to 1.91)	274(1 RCT)	⊕⊕⊕○ Moderate^d^	High-dose chemotherapy followed by autologous hematopoietic cell transplantation likely increases febrile neutropenia G3+.
Thrombocytopenia G3+	551 per 1,000	**849 per 1,000**(717 to 1,000)	**RR 1.54**(1.30 to 1.82)	274(1 RCT)	⊕⊕⊕○ Moderate^d^	High-dose chemotherapy followed by autologous hematopoietic cell transplantation likely increases thrombocytopenia G3+.
Nausea and vomiting G3+	125 per 1,000	**413 per 1,000**(254 to 673)	**RR 3.30**(2.03 to 5.38)	274(1 RCT)	⊕⊕○○ Low^e^	High-dose chemotherapy followed by autologous hematopoietic cell transplantation may result in a large increase in nausea and vomiting G3+.
Diarrhea G3+	15 per 1,000	**138 per 1,000**(33 to 580)	**RR 9.36**(2.22 to 39.42)	274(1 RCT)	⊕⊕○○ Low^e^	High-dose chemotherapy followed by autologous hematopoietic cell transplantation may result in a large increase in diarrhea G3+.
Mucositis G3+	22 per 1,000	**362 per 1,000**(116 to 1,000)	**RR 16.43**(5.25 to 51.39)	274(1 RCT)	⊕⊕○○ Low^e^	High-dose chemotherapy followed by autologous hematopoietic cell transplantation may result in a large increase in mucositis G3+.
Death due to toxicity	29 per 1,000	**65 per 1,000**(21 to 207)	**RR 2.22**(0.70 to 7.03)	274(1 RCT)	⊕⊕○○ Low^f^	High-dose chemotherapy followed by autologous hematopoietic cell transplantation may increase death due to toxicity.
Any chronic toxicity—not reported						No information was provided for any chronic toxicity.
*The risk in the intervention group (and its 95% confidence interval) is based on the assumed risk in the comparison group and the relative effect of the intervention (and its 95%CI).CI, confidence interval; HR, hazard ratio; RR, risk ratio.						
GRADE Working Group grades of evidence High certainty: we are very confident that the true effect lies close to that of the estimate of the effect. Moderate certainty: we are moderately confident in the effect estimate: the true effect is likely to be close to the estimate of the effect, but there is a possibility that it is substantially different. Low certainty: our confidence in the effect estimate is limited: the true effect may be substantially different from the estimate of the effect. Very low certainty: we have very little confidence in the effect estimate: the true effect is likely to be substantially different from the estimate of effect.						

Explanations:

a OS: As per RoB2, the IT94 trial was judged to be at high risk of bias due to deviations from the intended intervention that arose due to experimental context.

b EFS: According to RoB2, the IT94 trial was judged to be at high risk of bias due to deviations from the intended intervention that arose due to experimental context, bias due to missing data, and detection bias.

c Response rate outcomes: According to RoB2, the IT94 trial was judged to be at high risk of bias due to deviations from the intended intervention and detection bias. 95%CI crosses the line of no effect.

d Hematological acute toxicity: high risk of detection bias.

e Gastrointestinal acute toxicity: high risk of performance (especially nausea and diarrhea) and detection bias.

f Wide 95%CI due to few events.

### Primary outcome

#### Overall survival

Of 280 patients, 263 were evaluated for OS, considering data from the IT94 trial. After a median follow-up of 45 months, single HDCT followed by AHCT may have little to no effect on OS of men with relapsed GCTs (HR 0.98, 95%CI 0.68 to 1.42; p = 0.916; very low-certainty evidence).

**Figure 5:**
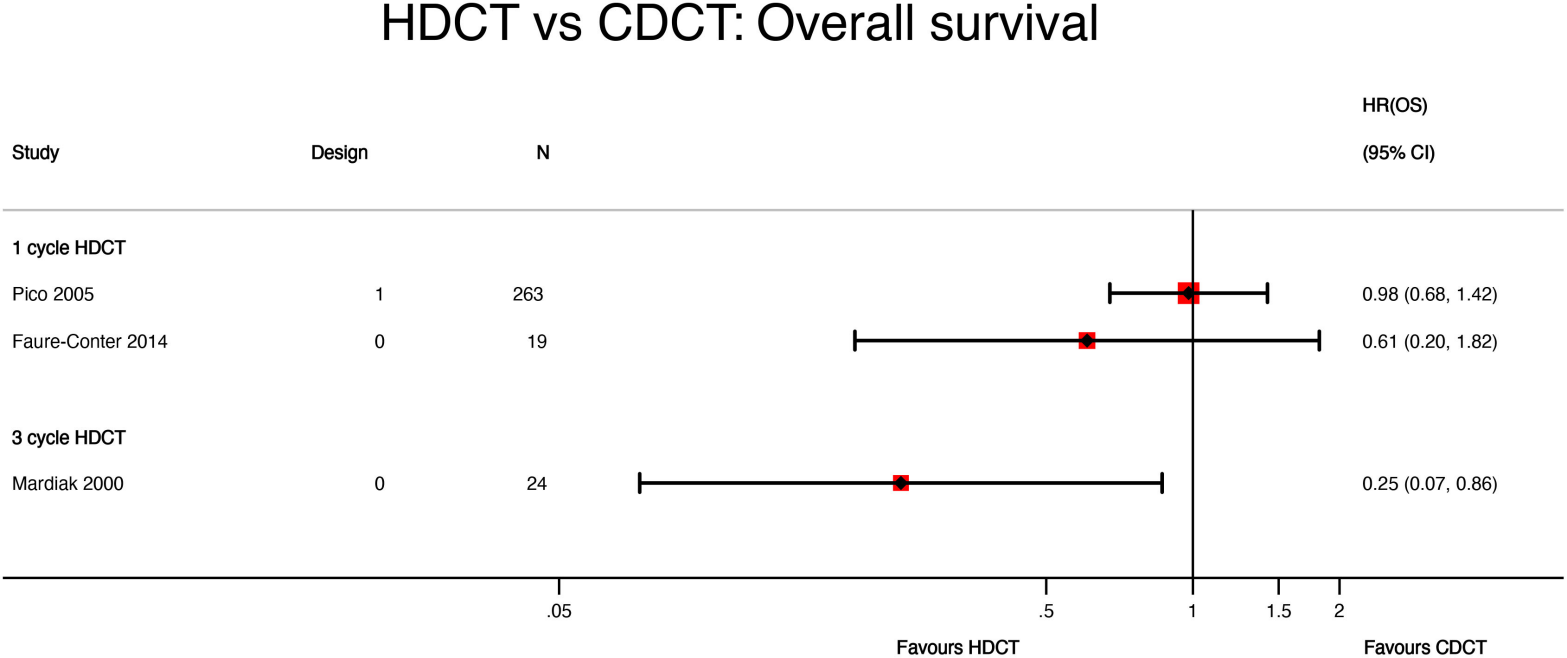
HDCT vs. CDCT: overall survival. HDCT, high-dose chemotherapy; CDCT, conventional-dose chemotherapy.

Mardiak’s (HR 0.25, 95%CI 0.07 to 0.86) and Faure-Conter’s (HR 0.61, 95%CI 0.20 to 1.82) studies showed contrasting and imprecise results, which may be explained by the number of cycles of high-dose chemotherapy administered in each study, patient’s characteristics, and the small sample size (**Figure 5** and **Table 3**).

**Table 3:** Overall survival based on data provided by non-randomized controlled trials^a^.

Study	HDCT n/N	CDCT n/N	lnHR	selnHR	HR (OS)	95%CI	p-Value
**Mardiak 2000 (** 32)	2/11	8/13	−1.38	0.63	0.25	0.07 to 0.86	0.03^b^
**Faure-Conter 2014 (** 33)	6/10	7/9	−0.5	0.56	0.61	0.20 to 1.82	0.37^c^

HDCT, high-dose chemotherapy; CDCT, conventional-dose chemotherapy; HR, hazard ratio; OS, overall survival.

a Hazard ratio calculations spreadsheet was used to facilitate the estimation of lnHRs and selnHRs from included studies ^11^.

b Mardiak's study provided p-values for 1-year (p = 0.03) and 2-year OS (p = 0.6). We were only able to extract data (n/N) for 1-year assessment.

c p-Value was supplied for abstract publication of Faure-Conter's study.^194^.

The lack of granularity of time-to-event data reported by Mardiak’s and Faure-Conter’s studies and their critical risk of bias impeded us from conducting a meta-analysis of OS.

### Secondary outcomes

The included studies did not provide data on QoL, PFS, and chronic toxicities.

#### Event-free survival

Considering only data supplied by the IT94 study, 263 out of 280 patients were evaluable for EFS. After a median follow-up of 45 months, single HDCT followed by AHCT may improve the EFS of men with relapsed GCTs (HR 0.80, 95%CI 0.59 to 1.10; p = 0.169; low-certainty evidence).

Mardiak’s study did not report data on EFS. On the contrary, Faure-Conter’s trial did display EFS information. However, the lack of detail on time-to-event data hindered us from obtaining EFS for experimental and control arms.

#### Response rate

Of 280 patients from the IT94 trial, 247 were evaluated for response rate (**Table 4**). Single HDCT followed by AHCT may result in little to no difference in the overall response rate (ORR) of men with relapsed GCTs (RR 1.03, 95%CI 0.84 to 1.26; p = 0.775; low-certainty evidence). Additionally, single HDCT with AHCT may not reduce failure of men with relapsed GCTs (RR 0.96, 95%CI 0.71 to 1.30; p = 0.775; low-certainty evidence). For further details about other response rate outcomes, see **Table 2**.

**Table 4:** Response rate outcomes only considering data from Pico 2005 (13).

Type of response	HDCT n/N	CDCT n/N	RR	95%CI	p-Value
**ORR**	76/125	72/122	1.03	0.84 to 1.26	0.78
**Failure**	49/125	50/122	0.96	0.71 to 1.30	0.78
**CR**	53/125	51/122	1.01	0.76 to 1.36	0.92
**PR plus normal tumor markers**	23/125	21/122	1.07	0.63 to 1.83	0.81

HDCT, high-dose chemotherapy; CDCT, conventional-dose chemotherapy; ORR, overall response rate; CR, complete response; PR, partial response.

Mardiak’s and Faure-Conter’s studies supplied data to explore response rate outcomes further. Considering the limitations of data reported from those trials, we found no evidence that HDCT followed by AHCT improves response rate outcomes, including complete response (CR), ORR, and failure (**Table 5**).

**Table 5:** Response rate considering data from Mardiak (2000) (32) and Faure-Conter (2014) (33).

Study	Type of response	HDCT n/N	CDCT n/N	RR	95%CI
**Mardiak (2000) (** 32)	ORR	7/11	6/14	1.48	0.70 to 3.15
	Failure	4/11	8/14	0.64	0.26 to 1.57
	CR	6/11	3/14	2.55	0.81 to 7.95
**Faure-Conter (2014) (** 33)	CR	5/10	2/9	2.25	0.57 to 8.86

HDCT, high-dose chemotherapy; CDCT, conventional-dose chemotherapy; ORR, overall response rate; CR, complete response.

**Figure 6:**
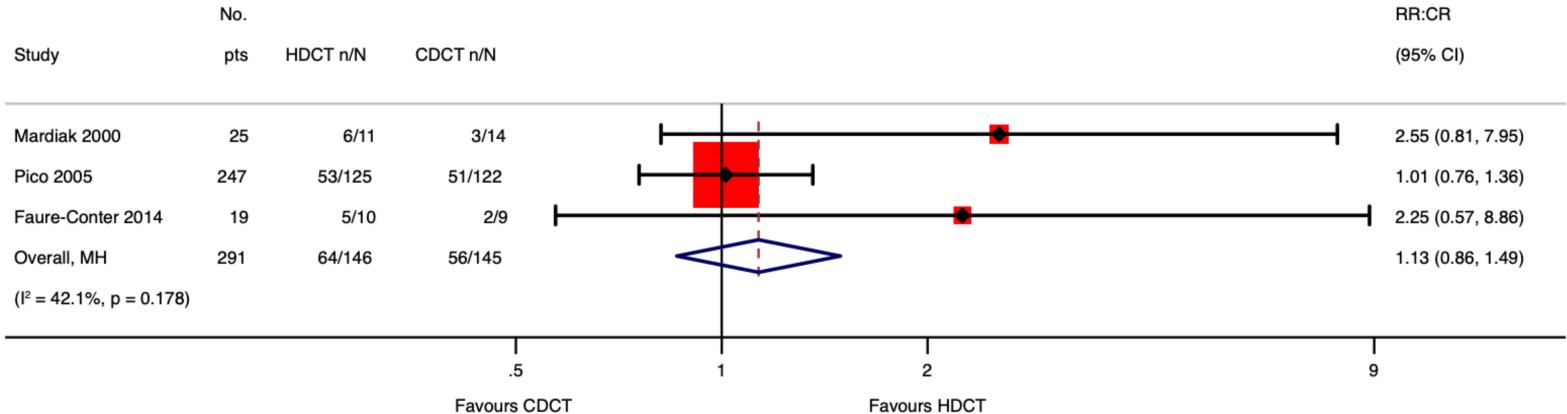
Meta-analysis of pooled estimates of complete response.

**Figure 7:**
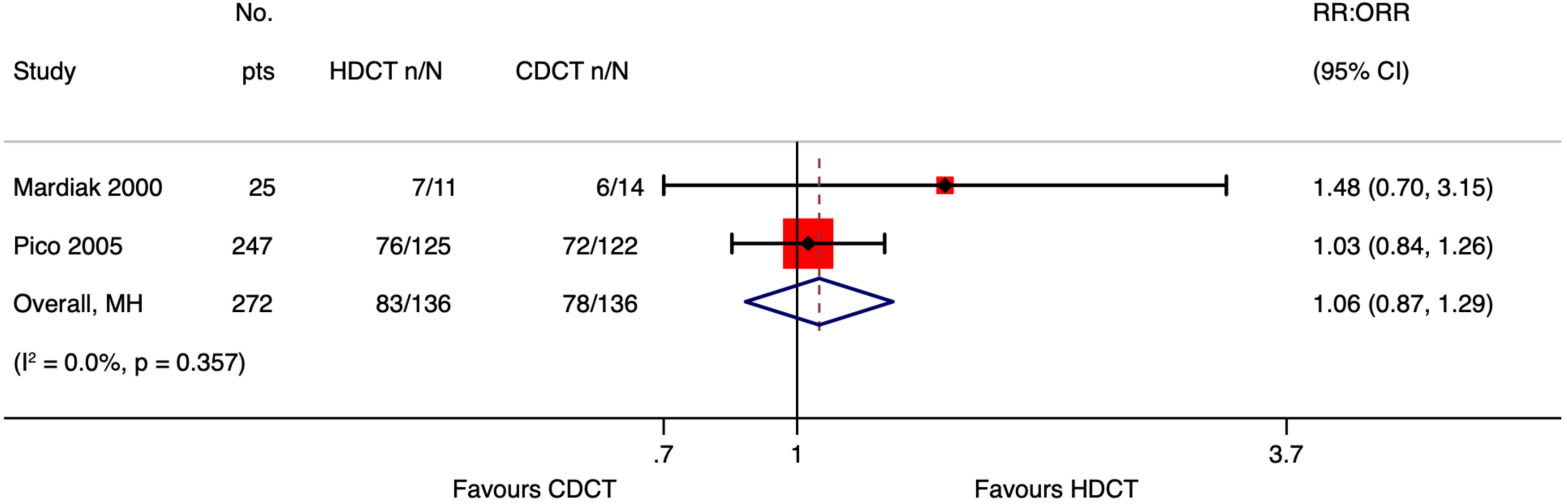
Meta-analysis of pooled estimates of overall response rate.

**Figure 8:**
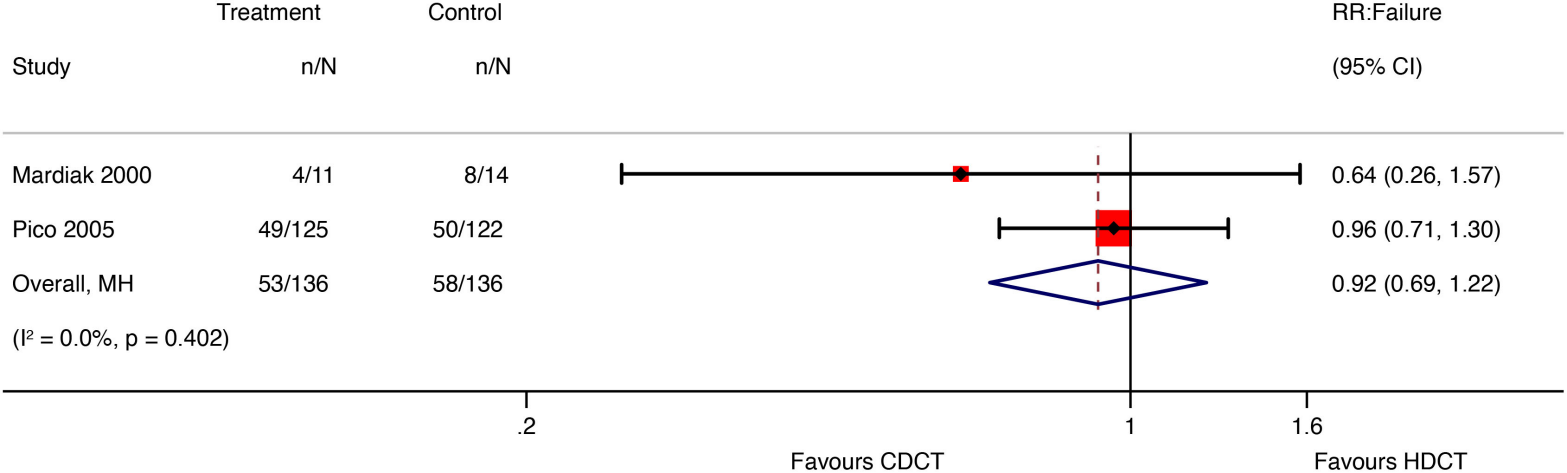
Meta-analysis of pooled estimates of failure.

See meta-analyses of complete response, overall response rate, and failure considering all included studies (**Figures 6**–**8**).

#### Acute toxicities

The included studies did not comprehensibly report acute AEs. The IT94 trial only reported some severe and life-threatening (G3≥) acute hematological and gastrointestinal toxicities. Mardiak’s study described acute hematological AEs and mentioned that non-hematological toxicities were rare during the study (e.g., two patients developed ischemic heart disease, and one patient had thrombophlebitis). Regarding Faure-Conter’s trial, the authors did not characterize acute toxicities whatsoever.

Acute toxicities were evaluated as per ITT and protocol analyses (see **Supplementary Tables S4** and **S5** in the **Supplementary Material**).

#### Acute hematological toxicity

Of 280 patients from the IT94 study, 274 were evaluable for acute hematological toxicities. Single HDCT followed by AHCT probably results in little to no difference in neutropenia ≥ G3 of men with relapsed GCTs (RR 1.06, 95%CI 0.98 to 1.14; p = 0.134; moderate-certainty evidence). However, single HDCT with AHCT likely increases febrile neutropenia ≥ G3 (RR 1.57, 95%CI 1.30 to 1.91; p < 0.001; moderate-certainty evidence) as well as thrombocytopenia ≥ G3 (RR 1.54, 95%CI 1.30 to 1.82; p < 0.001; moderate-certainty evidence) of men with relapsed GCTs.

**Figure 9:**
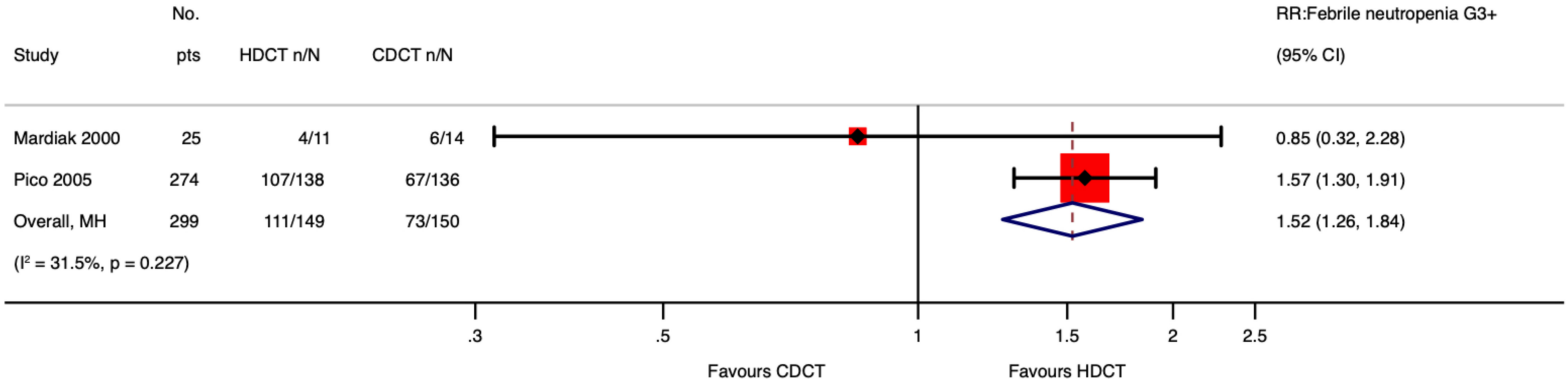
Meta-analysis of pooled estimates of febrile neutropenia ≥ G3.

**Figure 10:**
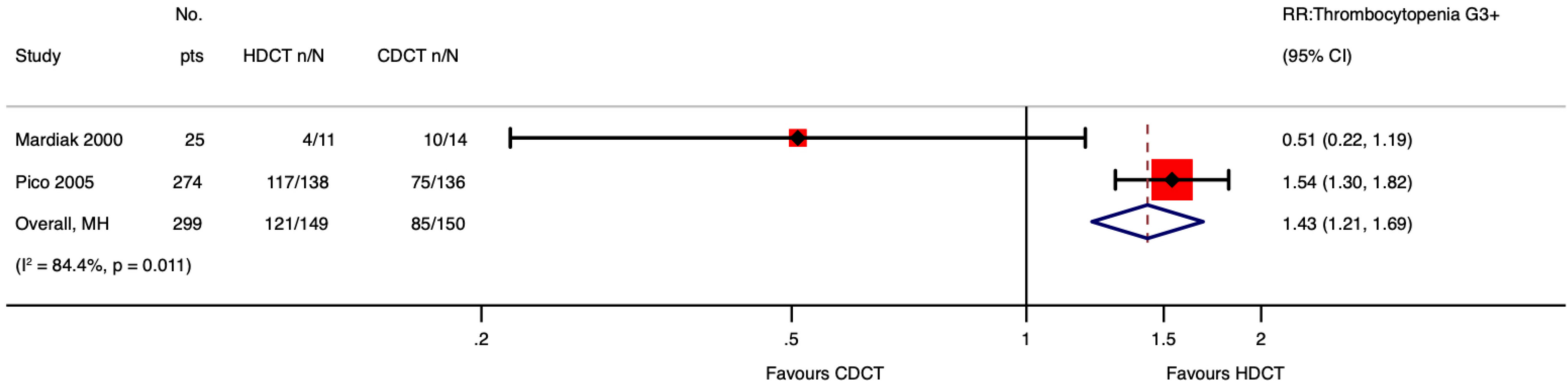
Meta-analysis of pooled estimates of thrombocytopenia ≥ G3.

Mardiak’s study found no evidence that HDCT, followed by AHCT, increases acute hematological toxicities of patients with GCTs (see **Supplementary Table S6** in the **Supplementary Material**). This is probably explained by the fact that the control arm did not receive the standard dose of VIP but 1.6 VIP (i.e., etoposide 600 mg/m^2^, ifosfamide 8,000 mg/m^2^, and cisplatin 100 mg/m^2^). See Meta-Analysis of Febrile Neutropenia and Thrombocytopenia considering all included studies (**Figures 9**, **10**).

#### Acute gastrointestinal toxicity

Of 280 patients from the IT94 trial, 274 were evaluable for acute gastrointestinal toxicities. Single HDCT followed by AHCT may result in a large increase in nausea/vomiting ≥ G3 (RR 3.30, 95%CI 2.03 to 5.38; p < 0.001; low-certainty evidence), diarrhea ≥ G3 (RR 9.36, 95%CI 2.22 to 39.42; p = 0.002; low-certainty evidence), and mucositis ≥ G3 (RR 16.43, 95%CI 5.25 to 51.39; p < 0.001; low-certainty evidence) of men with relapsed GCTs.

#### Death due to toxicity

The IT94 trial reported 9/138 deaths due to toxicities in the experimental arm and 4/136 deaths in the control arm. The causes of death due to toxicity can be found in **Supplementary Table S7** in the **Supplementary Material**.

Based on data from Pico’s trial, we concluded that single HDCT followed by AHCT may increase death due to toxicity of men with relapsed GCTs (RR 2.22, 95%CI 0.70 to 7.03; p = 0.176; low-certainty evidence).

Mardiak’s and Faure-Conter’s studies reported one death each. In the first study, the patient died due to toxicity (i.e., septic shock) during the first cycle of 1.6 VIP. On the contrary, one patient died of renal and myocardial acute failure after receiving CarboPEC in Faure-Conter’s trial.

## Discussion

### Summary of main results

The main objective of this SR and meta-analysis was to evaluate the efficacy of HDCT followed by AHCT versus CDCT in improving OS in men with relapsed or refractory GCTs. Three out of four studies were used to assess prespecified outcomes: one randomized (13) and two non-randomized prospective controlled trials (32, 33).

The IT94 trial (13) enrolled 280 participants, 263 of whom had data available for OS analysis. This study compared VIP/VeIP for four cycles with VIP/VeIP for 3 cycles, followed by one cycle of CarboPEC with AHCT. The IT94 study excluded patients with refractory disease. Based on the data provided by this study, we concluded that single HDCT followed by AHCT may have little to no effect on OS in relapsed GCT patients.

Non-randomized trials revealed contradictory results. Mardiak’s trial used three cycles of HDCT followed by AHCT. It showed that HDCT improves survival compared with CDCT. However, the OS benefit was lost at the 2-year assessment. On the contrary, Faure-Conter’s trial did not show statistical evidence that single HDCT improves OS in patients with GCTs.

Data from the IT94 trial (13) were utilized to produce the main conclusions about EFS, response rate, and acute toxicities. However, we conducted additional exploratory analysis using data from the included non-randomized studies (32, 33).

Based on the IT94 trial, we determined that single HDCT followed by AHCT may improve the EFS of men with relapsed GCTs. No significant differences were found in ORR and failure comparing single HDCT followed by AHCT with CDCT. Exploring the impact of data from included no-randomized trials on ORR and failure, we found no evidence that HDCT followed by AHCT improves CR or decreases failure.

As for acute toxicity ≥ G3, 274 out of 280 participants had data accessible for analysis. We concluded that single HDCT followed by AHCT likely increases the risk of developing febrile neutropenia and thrombocytopenia and may result in a large increase in nausea/vomiting, diarrhea, and mucositis. Exploring the influence of data from included no-randomized trials on febrile neutropenia and thrombocytopenia, we found that our conclusions did not change much. Finally, we also found that single HDCT followed by AHCT may increase death due to toxicity.

The conclusion about the primary outcome was supported by very-low-certainty evidence; thus, it should be interpreted cautiously. All secondary outcomes were exploratory and, therefore, are only hypothesis-generating.

### Quality of the evidence

The certainty of evidence was very low for OS due to study limitations and imprecision. Despite this, the IT94 trial provides the best available prospective evidence comparing the efficacy of single HDCT followed by AHCT with CDCT in men with relapsed GCTs.

The certainty of evidence-supported secondary outcomes such as EFS, response rate, acute gastrointestinal toxicity, and death due to toxicity was rated as low due to trial limitations and imprecision. On the contrary, due to study limitations, we rated the certainty of evidence for neutropenia, febrile neutropenia, and thrombocytopenia as moderate.

Despite that we included two non-randomized prospective controlled trials in our systematic review, data from these studies were not utilized to build the main conclusions. According to the ROBINS-I tool, the risk of bias in Mardiak’s and Faure-Conter’s studies was considered critical. Additionally, Mardiak’s trial had a small sample size (n = 25), included people with treatment-naïve GCTs (n = 9), and did not provide sufficient data to calculate time-to-event outcomes for relapsed/refractory GCT population. Faure-Conter’s trial also had a small sample size (n = 19) and included male and female infants (n = 9) as well as participants with ovarian and sacrococcygeal primary sites.

Finally, it is worth noting that included studies used HDCT and CDCT regimens, which are no longer utilized at high-volume cancer centers with experience in the management of GCTs (10, 17).

In view of the very-low-certainty evidence supporting OS, the current body of prospective data does not allow a definitive conclusion.

### Potential biases in the review process

The results of this SR provide a clear overview of the currently available prospective data regarding the value of HDCT with AHCT compared to CDCT in men with relapsed GCTs. We used a broad search strategy to identify eligible studies, searching three citation databases, two trial registers, and conference proceedings and checking the reference lists of previous reviews. We also considered non-English studies and used Google Translate when required. However, although it is unlikely that we missed eligible randomized controlled trials, we cannot be sure regarding non-randomized prospective controlled studies due to the search strategy utilized, issues related to indexing this type of study, and the quality of reporting. Regardless of these problems, it is improbable that we had missed a non-randomized prospective controlled study with a low risk of bias. In addition, we were not able to find updated data from included studies. Other limitations were that only one reviewer performed the screening of the studies and assessed the certainty of the evidence.

In addition, another source of potential bias was the decision to group different regimens of HDCT and CDCT in one experimental and one control arm, respectively. We made this pragmatic choice with the aim of conducting a subgroup analysis to further explain this source of variability. Nonetheless, we did not perform a meta-analysis of OS for the reason mentioned above.

### Agreements and disagreements with other studies or reviews

As we stated above, the aim of our systematic review was to assess the efficacy of high-dose chemotherapy followed by autologous hematopoietic cell transplantation versus conventional-dose chemotherapy in improving survival and other patient-important outcomes. Compared with previous reviews, ours was focused on summarizing the best available prospective evidence and addressing outcomes such as quality of life and toxicities.

The only available phase III randomized controlled trial (i.e., IT94 study), which compared CDCT with HDCT followed AHCT, failed to show a significant survival benefit from HDCT. Single-center studies conducted at high-volume academic cancer centers did show promising results utilizing HDCT with AHCT in this population (16, 17). Along the same lines, Lorch and colleagues found that HDCT, given as the first salvage therapy, improved PFS and OS in patients with relapsed/refractory germ cell tumors compared with CDCT regardless of IPFSG prognostic category except among low-risk patients (35). In contrast, a recent retrospective study found that patients with favorable and unfavorable-risk disease as per Memorial Sloan Kettering Cancer Center (MSKCC) criteria can also achieve durable responses with initial salvage TIP (36). These results show that experience matters, perhaps reflecting a better selection of patients, surgical expertise in managing residual mass, and the ability to safely deliver HDCT followed by AHCT. It is well known that death rates due to HDCT-related toxicity are largely related to expertise, as shown by Lorch et al. (35).

Systematic reviews have been published on this topic so far. They have shown results in keeping with our findings. For instance, Husnain et al. (18) failed to show any significant results for overall survival in patients with relapsed/refractory germ cell tumors treated with HDCT with AHCT. Petrelli and colleagues (22) found comparable efficacy when CDCT and HDCT were used as salvage therapies in relapsed/refractory germ cell tumors. Interestingly, Bin Riaz et al. (19) established that a single cycle of HDCT with AHCT does not improve survival compared with CDCT. However, the authors did find that two or three cycles of HDCT play a positive role in this population.

## Authors’ conclusions

### Implications for practice

There is insufficient prospective evidence to support or refute the proposal that HDCT with AHCT improves outcomes in men with relapsed or refractory GCTs.

### For clinicians working at high-volume cancer centers

If HDCT with AHCT is considered essential to treat people with relapsed/refractory GCTs, the choice should be made by experienced clinicians, taking into consideration the very-low-certainty evidence on OS and potential severe toxicities associated with this treatment. In addition, they must discuss with their patients the unknown impact of HDCT on QoL and long-term toxicities.

### For clinicians working at low-volume/community cancer centers

If salvage chemotherapy is considered essential to treat people with relapsed/refractory GCTs, clinicians should make a referral to a high-volume cancer center.

### Implications for research

#### General implications

This review revealed a lack of high-quality prospective research in the relapsed/refractory GCT population. However, the IT94 and TIGER trials show that it is possible to conduct phase III randomized controlled trials in a rare patient population.

We believe there is still room for an SR and meta-analysis of this topic. When the TIGER trial is published, a network meta-analysis using individual participant data should be performed comparing CDCT, single, and sequential HDCT to better understand the role of adding further cycles of HDCT in the efficacy of this therapy.

## Data Availability

The raw data supporting the conclusions of this article will be made available by the authors, without undue reservation.
