# Supplementary material for: High-dose chemotherapy as initial salvage chemotherapy in patients with relapsed or refractory testicular cancer: a systematic review and meta-analysis
Source: Front Oncol. 2024 Oct 1;14:1437574. doi: 10.3389/fonc.2024.1437574 (PMC11473300; doi:10.3389/fonc.2024.1437574)
Supplement: Supplementary file 1 [file DataSheet1.docx]

**Table S1** Characteristics of the excluded studies

| **Year** | **Author** | **Reason for exclusion** |
| --- | --- | --- |
| 1974 | Buckner ^24^ | No available abstract and full text |
| 1979 | DeWys ^25^ | Wrong intervention |
| 1981 | Blijham ^26^ | Wrong study design |
| 1986 | Stoter ^27^ | Wrong intervention |
| 1987 | Ozols ^28^ | Wrong patient population |
| 1988 | Ghosn ^29^ | Wrong study design |
| 1988 | Mulder ^30^ | Wrong study design |
| 1988 | Ozols ^31^ | Wrong patient population |
| 1989 | Nichols ^32^ | Wrong study design |
| 1991 | Broun ^33^ | Wrong study design |
| 1991 | Rosti ^34^ | Wrong study design |
| 1991 | Elias ^35^ | Wrong study design |
| 1992 | Broun ^36^ | Wrong study design |
| 1992 | Rosti ^37^ | Wrong study design |
| 1992 | Rodenhuis ^38^ | Wrong study design |
| 1992 | Santana ^39^ | Wrong study design |
| 1992 | Droz ^40^ | Wrong study design |
| 1992 | Motzer ^41^ | Wrong comparator |
| 1992 | Nichols ^42^ | Wrong study design |
| 1992 | Guimaraes ^43^ | Wrong study design |
| 1992 | Rosti ^44^ | Wrong study design |
| 1993 | Barnett ^45^ | Wrong study design |
| 1993 | Motzer ^46^ | Wrong study design |
| 1993 | Chevreau ^47^ | Wrong patient population |
| 1994 | Broun ^48^ | Wrong study design |
| 1994 | Siegert ^49^ | Wrong study design |
| 1995 | Lotz ^50^ | Wrong study design |
| 1995 | Rodenhuis ^51^ | Wrong study design |
| 1995 | Lampe ^52^ | Wrong study design |
| 1995 | Beyer ^53^ | Wrong comparator |
| 1995 | Takeda ^54^ | Wrong study design |
| 1995 | Ladenstein ^55^ | Wrong study design |
| 1996 | Van Warmerdam ^56^ | Wrong study design |
| 1996 | Farhat ^57^ | Wrong study design |
| 1996 | Motzer ^58^ | Wrong study design |
| 1996 | Margolin ^59^ | Wrong study design |
| 1996 | Fondazione IRCCS Istituto Nazionale dei Tumori ^60^ | Wrong patient population |
| 1997 | Beyer ^61^ | Wrong study design |
| 1997 | Broun ^62^ | Wrong study design |
| 1997 | Graham ^63^ | Wrong study design |
| 1997 | Motzer ^64^ | Wrong patient population |
| 1997 | Memorial Sloan Kettering Cancer Center ^65^ | Wrong study design |
| 1998 | Mandanas ^66^ | Wrong study design |
| 1998 | Papadopoulos ^67^ | Wrong study design |
| 1998 | Kanfer ^68^ | Wrong study design |
| 1998 | Lyttelton ^69^ | Wrong study design |
| 1999 | Rodenhuis ^70^ | Wrong study design |
| 1999 | Shamash ^71^ | Wrong study design |
| 1999 | Hara ^72^ | Wrong study design |
| 1999 | Gohji ^73^ | Wrong study design |
| 1999 | Nakagawa ^74^ | Wrong patient population |
| 1999 | Bokemeyer ^75^ | Wrong patient population |
| 1999 | European Organisation for Research ^76^ | Wrong patient population |
| 1999 | NCT00002508 ^77^ | Wrong study design |
| 1999 | NCT00002558 ^78^ | Wrong study design |
| 1999 | NCT00002594 ^79^ | Wrong study design |
| 1999 | NCT00002596 ^80^ | Wrong patient population |
| 1999 | NCT00002931 ^81^ | Wrong study design |
| 1999 | NCT00002943 ^82^ | Wrong study design |
| 1999 | NCT00003173 ^83^ | Wrong study design |
| 1999 | NCT00003811 ^84^ | Wrong study design |
| 1999 | NCT00003852 ^84^ | Wrong study design |
| 2000 | Shamash ^85^ | Wrong study design |
| 2000 | Kollmannsberger ^86^ | Wrong study design |
| 2000 | Decatris ^87^ | Wrong study design |
| 2000 | Miyazaki ^88^ | Wrong study design |
| 2000 | Bhatia ^89^ | Wrong study design |
| 2000 | Motzer ^90^ | Wrong study design |
| 2000 | Porcu ^91^ | Wrong study design |
| 2000 | NCT00005952 ^92^ | Wrong study design |
| 2001 | Rick ^93^ | Wrong patient population |
| 2001 | Ayash ^94^ | Wrong study design |
| 2001 | Kohda ^95^ | Wrong study design |
| 2001 | Rick ^96^ | Wrong study design |
| 2001 | NCT00007813 ^97^ | Wrong study design |
| 2001 | NCT00025324 ^98^ | Wrong patient population |
| 2002 | Rick ^99^ | Wrong comparator |
| 2002 | Beyer ^100^ | Wrong study design |
| 2002 | Rick ^101^ | Wrong study design |
| 2002 | Bokemeyer ^102^ | Wrong study design |
| 2002 | Rosti ^103^ | Wrong study design |
| 2002 | NCT00047320 ^104^ | Wrong study design |
| 2003 | Bokemeyer ^105^ | Wrong patient population |
| 2003 | Schmoll ^106^ | Wrong study design |
| 2003 | Billmire ^107^ | Wrong patient population |
| 2003 | NCT00060255 ^108^ | Wrong study design |
| 2004 | De Giorgi ^109^ | Wrong study design |
| 2004 | Rosti ^110^ | Wrong patient population |
| 2004 | McNeish ^111^ | Wrong study design |
| 2004 | Cushing ^112^ | Wrong intervention |
| 2004 | Hartmann ^113^ | Wrong comparator |
| 2004 | Modak ^114^ | Wrong study design |
| 2004 | Assistance Publique Hôpitaux de Paris ^115^ | Wrong study design |
| 2005 | Lotz ^116^ | Wrong study design |
| 2005 | Margolin ^117^ | Wrong study design |
| 2005 | Nieto ^118^ | Wrong study design |
| 2005 | Jordan ^119^ | Wrong study design |
| 2006 | Banna ^120^ | Wrong study design |
| 2006 | Müller ^121^ | Wrong study design |
| 2006 | Fraser ^122^ | Wrong patient population |
| 2006 | Bajorin ^123^ | Wrong patient population |
| 2007 | Droz ^124^ | Wrong patient population |
| 2007 | Miki ^125^ | Wrong patient population |
| 2007 | Hartmann ^126^ | Wrong study design |
| 2007 | Kondagunta ^127^ | Wrong study design |
| 2007 | Lorch ^2^ | Wrong comparator |
| 2007 | Motzer ^128^ | Wrong patient population |
| 2007 | Einhorn ^129^ | Wrong study design |
| 2007 | EUCTR2006-006004-11-DE ^130^ | Wrong study design |
| 2007 | NCT00423852 ^131^ | Wrong study design |
| 2007 | NCT00432094 ^132^ | Wrong comparator |
| 2007 | NCT00436774 ^133^ | Wrong patient population |
| 2007 | NCT00467051 ^134^ | Wrong study design |
| 2007 | NCT00536601 ^135^ | Wrong study design |
| 2007 | NCT00551122 ^136^ | Wrong study design |
| 2008 | Laughton ^137^ | Wrong study design |
| 2008 | Oechsle ^138^ | Wrong study design |
| 2008 | Ozkaynak ^139^ | Wrong study design |
| 2009 | Agarwal ^140^ | Wrong study design |
| 2009 | Beyer ^141^ | Wrong study design |
| 2009 | Lotz ^142^ | Wrong study design |
| 2009 | JPRN-UMIN000002398 ^143^ | Wrong study design |
| 2009 | M.D. Anderson Cancer Center ^144^ | Wrong comparator |
| 2010 | Lorch ^145^ | Wrong study design |
| 2010 | Feldman ^146^ | Wrong study design |
| 2010 | Lorch ^147^ | Wrong patient population |
| 2010 | Fondazione IRCCS Istituto Nazionale dei Tumori ^148^ | Wrong study design |
| 2011 | Daugaard ^149^ | Wrong patient population |
| 2011 | Olofsson ^150^ | Wrong study design |
| 2011 | Asirwa ^151^ | Wrong study design |
| 2012 | Haugnes ^152^ | Wrong study design |
| 2012 | De Giorgi ^153^ | Wrong study design |
| 2012 | Hartmann ^154^ | Wrong patient population |
| 2012 | Carmichael ^155^ | Wrong study design |
| 2012 | NCT01505569 ^156^ | Wrong study design |
| 2013 | Suleiman ^157^ | Wrong study design |
| 2013 | NCT01966913 ^158^ | Wrong study design |
| 2014 | Selle ^159^ | Wrong study design |
| 2014 | Robertson ^160^ | Wrong study design |
| 2014 | Necchi ^161^ | Wrong study design |
| 2014 | Nieto ^162^ | Wrong study design |
| 2015 | Necchi ^163^ | Wrong patient population |
| 2015 | Nieto ^164^ | Wrong study design |
| 2015 | Feldman ^165^ | Wrong study design |
| 2015 | Goldman ^166^ | Wrong study design |
| 2015 | Necchi ^167^ | Wrong patient population |
| 2016 | De Filipp ^168^ | Wrong study design |
| 2016 | Egan ^169^ | Wrong study design |
| 2016 | Necchi ^170^ | Wrong study design |
| 2016 | DRKS00010317 ^171^ | Wrong patient population |
| 2016 | NCT02784054 ^172^ | Wrong study design |
| 2017 | Moeung ^173^ | Wrong study design |
| 2017 | Kalra ^174^ | Wrong study design |
| 2017 | Kegel ^175^ | Wrong study design |
| 2017 | Nieto ^176^ | Wrong comparator |
| 2018 | Gössi ^177^ | Wrong study design |
| 2018 | Kilari ^178^ | Wrong comparator |
| 2018 | Osorio ^179^ | Wrong study design |
| 2019 | Adra ^180^ | Wrong study design |
| 2019 | Callera ^181^ | Wrong study design |
| 2020 | Anouti ^182^ | Wrong patient population |
| 2020 | Adra ^183^ | Wrong study design |
| 2020 | Agrawal ^184^ | Wrong study design |
| 2020 | NCT04521946 ^185^ | Wrong patient population |
| 2020 | NCT04581265 ^186^ | Wrong study design |
| 2021 | Chevreau ^187^ | Wrong study design |
| 2021 | Adra ^188^ | Wrong patient population |
| 2022 | Madanchi ^189^ | Wrong study design |
| 2022 | Taza ^190^ | Wrong study design |
| 2022 | Ashkar ^191^ | Wrong patient population |
| 2022 | NCT05455918 ^192^ | Wrong study design |
| 2023 | Taza ^193^ | Wrong patient population |

**Tables S2A-E** Support for judgement of risk of bias assessment. Risk of bias assessment of IT94 trial. **A.** Overall survival. **B.** Event-free survival. **C.** Response rate. **D.** Toxicity (ITT analysis). **E.** Toxicity (Per-protocol analysis).

**Table S2A**

|  |  |  |
| --- | --- | --- |
| Outcome: Overall survival  ITT analysis |  |  |
| Bias | **Authors´ judgement** | **Support for judgement** |
| Bias arising from the randomization process | Low | Central randomization was used, ensuring random sequence generation and allocation concealment |
| Bias due to deviation from intended intervention | High | Open label trial. Obvious differences between interventions. There were deviations from the intended intervention that arose due to experimental context |
| Bias due to missing outcome data | Low | Assessment of an objective outcome (death due to any cause) |
| Bias in measurement of the outcome | Low | Assessment of an objective outcome |
| Bias in selection of the reported result | Low | Study report published in ClinicalTrials.gov.  No information was provided about secondary outcomes in the study report. However, we determined that the selection of the reported result was unlikely. |
| Overall bias | High |  |

**Table S2B**

|  |  |  |
| --- | --- | --- |
| Outcome: Event-free survival  ITT analysis |  |  |
| Bias | **Authors´ judgement** | **Support for judgement** |
| Bias arising from the randomization process | Low | Central randomization was used, ensuring random sequence generation and allocation concealment |
| Bias due to deviation from intended intervention | High | Open label trial. Obvious differences between interventions. There were deviations from the intended intervention that arose due to experimental context |
| Bias due to missing outcome data | High | Assessment of a subjective outcome. Several patients were withdrawn in each arm due to refractory disease |
| Bias in measurement of the outcome | High | No information about blinding of outcome assessors. Assessment of a subjective outcome |
| Bias in selection of the reported result | Low | Study report published in ClinicalTrials.gov.  Pre-planned methodology was consistent with what was actually done. |
| Overall bias | High |  |

**Table S2C**

|  |  |  |
| --- | --- | --- |
| Outcome: Response rate  ITT analysis |  |  |
| Bias | **Authors´ judgement** | **Support for judgement** |
| Bias arising from the randomization process | Low | Central randomization was used, ensuring random sequence generation and allocation concealment |
| Bias due to deviation from intended intervention | High | Open label trial. Obvious differences between interventions. There were deviations from the intended intervention that arose due to experimental context |
| Bias due to missing outcome data | Low | 247/280 were evaluable for overall response |
| Bias in measurement of the outcome | High | No information about blinding of outcome assessors. Assessment of a subjective outcome |
| Bias in selection of the reported result | Low | Study report published in ClinicalTrials.gov.  No information was provided about secondary outcomes in the study report. However, we determined that the selection of the reported result was unlikely. |
| Overall bias | High |  |

**Table S2D**

|  |  |  |
| --- | --- | --- |
| Outcome: Toxicity  ITT analysis |  |  |
| Bias | **Authors´ judgement** | **Support for judgement** |
| Bias arising from the randomization process | Low | Central randomization was used, ensuring random sequence generation and allocation concealment |
| Bias due to deviation from intended intervention | High | Open label trial. Obvious differences between interventions. There were deviations from the intended intervention that arose due to experimental context |
| Bias due to missing outcome data | Low | 274/280 were evaluable for toxicity |
| Bias in measurement of the outcome | High | No information about blinding of outcome assessors. Assessment of a subjective outcome |
| Bias in selection of the reported result | Low | Study report published in ClinicalTrials.gov.  No information was provided about secondary outcomes in the study report. However, we determined that the selection of the reported result was unlikely. |
| Overall bias | High |  |

**Table S2E**

|  |  |  |
| --- | --- | --- |
| Outcome: Toxicity  PP analysis |  |  |
| Bias | **Authors´ judgement** | **Support for judgement** |
| Bias arising from the randomization process | Low | Central randomization was used, ensuring random sequence generation and allocation concealment |
| Bias due to deviation from intended intervention | Some concerns | Open label trial. There were deviations from the intended intervention that arose due to only 71% of participant received HDCT (96/135 pts). Considering that both treatment arms are exactly the same until the 3^rd^ cycle, and just the 4^rd^ cycle was different between arms |
| Bias due to missing outcome data | Low | 274/280 patients were evaluable for toxicity. All patients who received the 4rd cycle had information available about toxicity. |
| Bias in measurement of the outcome | High | No information about blinding of outcome assessors. Assessment of a subjective outcome |
| Bias in selection of the reported result | Low | Study report published in ClinicalTrials.gov.  No information was provided about secondary outcomes in the study report. However, we determined that the selection of the reported result was unlikely. |
| Overall bias | High |  |

**Table S3A-E** Support for judgement of risk of bias assessment. Risk of bias assessment of non-randomized trials. **A.** Overall survival (Mardiak 2000). **B.** Overall survival (Faure-Conter 2014). **C.** Response rate (Mardiak 2000). **D.** Toxicity (ITT analysis) (Mardiak 2000). **E.** Toxicity (Per-protocol analysis) (Mardiak 200).

**Table S3A**

|  |  |  |
| --- | --- | --- |
| Outcome: Overall survival  ITT analysis |  |  |
| Bias | **Authors´ judgement** | **Support for judgement** |
| Bias due to confounding | Critical | Includes participants with different prognosis at baseline.  Authors did not use adequate statistical analysis to adjust for confounding. |
| Bias in selection of participants into the study | Critical | Includes 25 patients, the first 14 participants received 1.6 VIP, and based on tolerance, the remaining 11 patients were treated with 1.9 VIP followed by AHCT. |
| Bias in classification of interventions | Low | Defines interventions at the beginning of the study. |
| Bias due to deviations from intended interventions | Low | Deviations from intended intervention was not beyond what would be expected in usual practice |
| Bias due to missing data | Low | Since outcome of interest is death due to any causes, complete outcome data was probably achieved. |
| Bias in measurement of outcomes | Low | The outcome of interest is objective |
| Bias in selection of the reported result | Moderate | No available protocols. However, there was no clear indication of selection of the reported analysis from among multiple analyses. |
| Overall bias | Critical |  |

**Table S3B**

|  |  |  |
| --- | --- | --- |
| Outcome: Overall survival  ITT analysis |  |  |
| Bias | **Authors´ judgement** | **Support for judgement** |
| Bias due to confounding | Critical | Includes patients with different prognosis at baseline, and treated them according to their risk at baseline. Authors did not use appropriate statistical analysis to control for all important confounding. |
| Bias in selection of participants into the study | Critical | The study is part of the French TGM95 Protocol. The TGM95 included 273 patients with extracranial non-seminoma GCT. The first strategy used to treat GCT was the surgical removal of the tumor when it was possible. Patients were categorized in low, intermediate and high-risk depending on AFP levels and quality of surgery. Patients were treated according to their risk at baseline. In case of non-remission, progression during chemotherapy or recurrent non-seminoma GCT, salvage chemotherapy was recommended: intermediate-risk participants received CDCT and high-risk patients, HDCT with AHCT. Authors did not adjust for selection bias. |
| Bias in classification of interventions | Low | Defines interventions at the beginning of the study. |
| Bias due to deviations from intended interventions | Low | Deviations from intended intervention was not beyond what would be expected in usual practice |
| Bias due to missing data | Low | Since outcome of interest is death due to any causes, complete outcome data was probably achieved. |
| Bias in measurement of outcomes | Low | Outcome of interest is objective |
| Bias in selection of the reported result | Moderate | No available protocols. However, there was no clear indication of selection of the reported analysis from among multiple analyses. |
| Overall bias | Critical |  |

**Table S3C**

| Outcome: Response rate  ITT analysis |  |  |
| --- | --- | --- |
| Bias | **Authors´ judgement** | **Support for judgement** |
| Bias due to confounding | Critical | Includes participants with different prognosis at baseline.  Authors did not use adequate statistical analysis to adjust for confounding. |
| Bias in selection of participants into the study | Critical | Includes 25 patients, the first 14 participants received 1.6 VIP, and based on tolerance, the remaining 11 patients were treated with 1.9 VIP followed by AHCT. |
| Bias in classification of interventions | Low | Defines interventions at the beginning of the study. |
| Bias due to deviations from intended interventions | Low | Deviations from intended intervention was not beyond what would be expected in usual practice |
| Bias due to missing data | Low | Response rate data available from 25 patients. |
| Bias in measurement of outcomes | Serious | The outcome of interest is subjective |
| Bias in selection of the reported result | Moderate | No available protocols. However, there was no clear indication of selection of the reported analysis from among multiple analyses. |
| Overall bias | Critical |  |

**Table S3D**

|  |  |  |
| --- | --- | --- |
| Outcome: Toxicity  ITT analysis |  |  |
| Bias | **Authors´ judgement** | **Support for judgement** |
| Bias due to confounding | Critical | Includes participants with different prognosis at baseline.  Authors did not use adequate statistical analysis to adjust for confounding. |
| Bias in selection of participants into the study | Critical | Includes 25 patients, the first 14 participants received 1.6 VIP, and based on tolerance, the remaining 11 patients were treated with 1.9 VIP followed by AHCT. |
| Bias in classification of interventions | Low | Defines interventions at the beginning of the study. |
| Bias due to deviations from intended interventions | Low | Deviations from intended intervention was not beyond what would be expected in usual practice |
| Bias due to missing data | Low | Toxicity data available from all patients |
| Bias in measurement of outcomes | Moderate | The outcomes of interests are measured objectively however, their interpretation might be biased. |
| Bias in selection of the reported result | Moderate | No available protocols. However, there was no clear indication of selection of the reported analysis from among multiple analyses. |
| Overall bias | Critical |  |

**Table S3E**

|  |  |  |
| --- | --- | --- |
| Outcome: Toxicity  PP analysis |  |  |
| Bias | **Authors´ judgement** | **Support for judgement** |
| Bias due to confounding | Critical | Includes participants with different prognosis at baseline.  Authors did not use adequate statistical analysis to adjust for confounding. |
| Bias in selection of participants into the study | Critical | Includes 25 patients, the first 14 participants received 1.6 VIP, and based on tolerance, the remaining 11 patients were treated with 1.9 VIP followed by AHCT. |
| Bias in classification of interventions | Low | Defines interventions at the beginning of the study. |
| Bias due to deviations from intended interventions | Low | No evidence of lack of adherence to intervention |
| Bias due to missing data | Low | Toxicity data available from all patients |
| Bias in measurement of outcomes | Moderate | The outcomes of interests are measured objectively however, their interpretation might be biased |
| Bias in selection of the reported result | Moderate | No available protocols. However, there was no clear indication of selection of the reported analysis from among multiple analyses. |
| Overall bias | Critical |  |

**Table S4** Acute toxicities as per protocol analysis (Pico 2005).

| Toxicity G3 ≥^a^ | HDCT n/N | CDCT n/N | RR | 95%CI | p-value |
| --- | --- | --- | --- | --- | --- |
| Neutropenia | 98/98 | 68/110 | 1.61 | 1.39 to 1.87 | <0.001 |
| Febrile neutropenia | 93/98 | 25/110 | 4.18 | 2.95 to 5.91 | <0.001 |
| Thrombocytopenia | 98/98 | 46/110 | 2.38 | 1.91 to 2.96 | <0.001 |
| Nausea and vomiting | 47/98 | 4/110 | 13.19 | 4.93 to 35.28 | <0.001 |
| Diarrhea | 18/98 | 0/110 | 41.48 | 2.53 to 679.42 | 0.009 |
| Mucositis | 48/98 | 0/110 | 108.76 | 6.80 to 1740.58 | 0.001 |

^a^ Toxicity evaluation was conducted after receiving cycle number 4

**Table S5** Acute toxicities as per ITT analysis (Pico 2005).

| Toxicity G3 ≥ | HDCT n/N | CDCT n/N | RR | 95%CI | p-value |
| --- | --- | --- | --- | --- | --- |
| Neutropenia | 129/138 | 120/136 | 1.06 | 0.98 to 1.14 | 0.134 |
| Febrile neutropenia | 107/138 | 67/136 | 1.57 | 1.30 to 1.91 | <0.001 |
| Thrombocytopenia | 117/138 | 75/136 | 1.54 | 1.30 to 1.82 | <0.001 |
| Nausea and vomiting | 57/138 | 17/136 | 3.30 | 2.03 to 5.38 | <0.001 |
| Diarrhea | 19/138 | 2/136 | 9.36 | 2.22 to 39.42 | 0.002 |
| Mucositis | 50/138 | 3/136 | 16.43 | 5.25 to 51.39 | <0.001 |

**Table S6** Acute toxicities as per ITT analysis (Mardiak 2000).

| Toxicity G3 ≥ | HDCT n/N | CDCT n/N | RR | 95%CI | p-value |
| --- | --- | --- | --- | --- | --- |
| Leukopenia | 7/11 | 11/14 | 0.81 | 0.48 to 1.37 | 0.430 |
| Febrile neutropenia | 4/11 | 6/14 | 0.85 | 0.32 to 2.28 | 0.745 |
| Thrombocytopenia | 4/11 | 10/14 | 0.51 | 0.22 to 1.19 | 0.119 |
| Anemia | 0/11 | 3/14 | 0.18 | 0.01 to 3.13 | 0.238 |

**Table S7** Death due to toxicity (Pico 2005).

| HDCT (9 deaths) | CDCT (4 deaths) |
| --- | --- |
| Sepsis, renal failure and ARDS: 4 patients | Septic shock: 2 patients |
| Multi-organ failure: 2 patients | Neutropenia and thrombocytopenia: 1 patient |
| Brain hemorrhage: 1 patient | Heart failure: 1 patient |
| Diffuse alveolar hemorrhage: 1 patient |  |
| Complication arising from surgery: 1 patient |  |

**Appendix 1.**

**Search strategy for PubMed**

**#1 “Testicular Neoplasms"[Mesh]**

**#2 testicular malignancy [Title/Abstract]**

**#3 tumor of the testis [Title/Abstract]**

**#4 cancer of the testis [Title/Abstract]**

**#5 testicular cancer [Title/Abstract]**

**#6 germ cell tumor [Title/Abstract]**

**#7 extragonadal germ cell tumor [Title/Abstract]**

**#8 testicular germ cell tumor [Title/Abstract]**

**#9 mediastinal germ cell tumor [Title/Abstract]**

**#10 retroperitoneal germ cell tumor [Title/Abstract]**

**#11 intracranial germ cell tumor [Title/Abstract]**

**#12 "Neoplasms, Germ Cell and Embryonal"[Mesh]**

**#13 seminoma [Title/Abstract]**

**#14 “Seminoma” [Mesh]**

**#15 non-seminomatous [Title/Abstract]**

**#16 “Nonseminomatous germ cell tumor” [Supplementary Concept]**

**#17 yolk sac tumor [Title/Abstract]**

**#18 choriocarcinoma [Title/Abstract]**

**#19 embryonal carcinoma [Title/Abstract]**

**#20 teratoma [Title/Abstract]**

**#21 #1 OR #2 OR #3 OR #4 OR #5 OR #6 OR #7 OR #8 OR #9 OR #10 OR #11 OR #12 or #13 OR #14 OR #15 OR #16 OR #17 OR #18 OR #19 OR #20**

**#22 high dose chemotherapy [Title/Abstract]**

**#23 Bone Marrow Transplant* [Title/Abstract]**

**#24 "Bone Marrow Transplantation" [Mesh]**

**#25 carboplatin [Title/Abstract]**

**#26 “Carboplatin" [Mesh]**

**#27 etoposide [Title/Abstract]**

**#28 "Etoposide" [Mesh]**

**#29 cyclophosphamide [Title/Abstract]**

**#30 "Cyclophosphamide" [Mesh]**

**#31 ifosfamide [Title/Abstract]**

**#32 "Ifosfamide" [Mesh]**

**#33 alkylating agents [Title/Abstract]**

**#34 "Alkylating Agents" [Mesh]**

**#35 stem cell transplant* [Title/Abstract]**

**#36 "Stem Cell Transplantation"[Mesh]**

**#37 marrow ablative chemotherapy [Title/Abstract]**

**#38 myeloablative chemotherapy [Title/Abstract]**

#39 **#22 OR #23 OR #24 OR #25 OR #26 OR #27 OR #28 OR #29 OR #30 or #31 OR #32 OR #33 OR #34 OR #35 OR #36 OR #37 OR #38**

**#40 #31 AND #39**

Filters: **Clinical Trial, Clinical Trial Protocol, Clinical Trial, Phase I, Clinical Trial, Phase II, Clinical Trial, Phase III, Clinical Trial, Phase IV, Controlled Clinical Trial, Male, Adolescent: 13-18 years, Adult: 19+ years**

**Appendix 2.**

**Search strategy for EMBASE Ovid**

#1 testicular neoplasms.mp. or exp testis tumor/

#2 limit 1 to (human and clinical trial and "therapy (maximizes sensitivity)" and adult <18 to 64 years> and "humans only (removes records about animals)")

#3 testicular malignancy.mp. or exp testis cancer/

#4 limit 3 to (human and clinical trial and "therapy (maximizes sensitivity)" and article and adult <18 to 64 years> and "humans only (removes records about animals)")

#5 tumor of the testis.mp. or exp testis tumor/

#6 limit 5 to (human and clinical trial and "therapy (maximizes sensitivity)" and article and adult <18 to 64 years> and "humans only (removes records about animals)")

#7 cancer of the testis.mp. or exp testis cancer/

#8 limit 7 to (human and clinical trial and "therapy (maximizes sensitivity)" and article and adult <18 to 64 years> and "humans only (removes records about animals)")

#9 testicular cancer.mp. or exp testis cancer/

#10 limit 9 to (human and clinical trial and "therapy (maximizes sensitivity)" and article and adult <18 to 64 years> and "humans only (removes records about animals)")

#11 germ cell tumor.mp. or exp germ cell tumor/

#12 limit 11 to (human and clinical trial and "therapy (maximizes sensitivity)" and article and adult <18 to 64 years> and "humans only (removes records about animals)")

#13 exp retroperitoneal tumor/ or exp germ cell tumor/ or exp mediastinum tumor/ or extragonadal germ cell tumor.mp.

#14 limit 13 to (human and clinical trial and "therapy (maximizes sensitivity)" and article and adult <18 to 64 years> and "humans only (removes records about animals)")

#15 testicular germ cell tumor.mp. or exp germ cell tumor/ or exp testicular germ cell tumor/ or exp testis tumor/ or exp testis cancer/ or exp seminoma/

#16 limit 15 to (human and clinical trial and "therapy (maximizes sensitivity)" and article and adult <18 to 64 years> and "humans only (removes records about animals)")

#17 exp teratoma/ or mediastinal germ cell tumor.mp. or exp germ cell tumor/ or exp mediastinal germ cell tumor/ or exp mediastinum cancer/ or exp mediastinum tumor/

#18 limit 17 to (human and clinical trial and "therapy (maximizes sensitivity)" and article and adult <18 to 64 years> and "humans only (removes records about animals)")

#19 exp germ cell tumor/ or exp retroperitoneal tumor/ or exp retroperitoneal cancer/ or retroperitoneal germ cell tumor.mp. or exp seminoma/

#20 limit 19 to (human and clinical trial and "therapy (maximizes sensitivity)" and article and adult <18 to 64 years> and "humans only (removes records about animals)")

#21 exp teratoma/ or exp intracranial tumor/ or exp germ cell tumor/ or intracranial germ cell tumor.mp.

#22 limit 21 to (human and clinical trial and "therapy (maximizes sensitivity)" and article and adult <18 to 64 years> and "humans only (removes records about animals)")

#23 exp choriocarcinoma/ or intracraneal germ cell tumor.mp.

#24 limit 23 to (human and clinical trial and "therapy (maximizes sensitivity)" and article and adult <18 to 64 years> and "humans only (removes records about animals)")

#25 exp classical seminoma/ or exp seminoma/ or exp spermatocytic seminoma/ or exp germ cell tumor/ or exp mediastinal seminoma/ or seminoma.mp. or exp non seminomatous germinoma/

#26 limit 25 to (human and clinical trial and "therapy (maximizes sensitivity)" and article and adult <18 to 64 years> and "humans only (removes records about animals)")

#27 exp testis tumor/ or exp non seminomatous germinoma/ or exp germ cell tumor/ or non seminomatous.mp. or exp testis cancer/

#28 limit 27 to (human and clinical trial and "therapy (maximizes sensitivity)" and article and adult <18 to 64 years> and "humans only (removes records about animals)")

#29 exp testis teratoma/ or teratoma/ or exp intracranial teratoma/ or exp malignant teratoma/ or exp mediastinal teratoma/ or teratoma.mp.

#30 limit 29 to (clinical trial and "therapy (maximizes sensitivity)" and article and adult <18 to 64 years> and "humans only (removes records about animals)")

#31 yolk sac tumor.mp. or exp yolk sac tumor/

#32 limit 31 to (human and clinical trial and "therapy (maximizes sensitivity)" and article and adult <18 to 64 years> and "humans only (removes records about animals)")

#33 choriocarcinoma.mp. or exp choriocarcinoma/ or exp gastric choriocarcinoma/ or exp pulmonary choriocarcinoma/ or exp testicular choriocarcinoma/ or exp mediastinal choriocarcinoma/ or exp metastatic choriocarcinoma/

#34 limit 33 to (human and clinical trial and "therapy (maximizes sensitivity)" and article and adult <18 to 64 years> and "humans only (removes records about animals)")

#35 embryonal carcinoma.mp. or exp embryonal carcinoma/ or exp testis cancer/

#36 limit 35 to (human and clinical trial and "therapy (maximizes sensitivity)" and article and adult <18 to 64 years> and "humans only (removes records about animals)")

**#37 2 or 4 or 6 or 8 or 10 or 12 or 14 or 16 or 18 or 20 or 22 or 24 or 26 or 28 or 30 or 32 or 34 or 36**

#38 high dose chemotherapy.mp.

#39 limit 38 to (human and clinical trial and "therapy (maximizes sensitivity)" and article and adult <18 to 64 years> and "humans only (removes records about animals)")

#40 exp bone marrow transplantation/ or bone marrow transplant*.mp.

#41 limit 40 to (human and clinical trial and "therapy (maximizes sensitivity)" and article and adult <18 to 64 years> and "humans only (removes records about animals)")

#42 carboplatin.mp. or exp carboplatin/

#43 limit 42 to (human and clinical trial and "therapy (maximizes sensitivity)" and article and adult <18 to 64 years> and "humans only (removes records about animals)")

#44 exp etoposide/

#45 limit 44 to (human and clinical trial and "therapy (maximizes sensitivity)" and article and adult <18 to 64 years> and "humans only (removes records about animals)")

#46 exp cyclophosphamide/ or cyclophosphamide.mp.

#47 limit 46 to (human and clinical trial and "therapy (maximizes sensitivity)" and article and adult <18 to 64 years> and "humans only (removes records about animals)")

#48 ifosfamide.mp. or exp ifosfamide plus mesna/ or exp ifosfamide/

#49 limit 48 to (human and clinical trial and "therapy (maximizes sensitivity)" and article and adult <18 to 64 years> and "humans only (removes records about animals)")

#50 alkylating agents.mp. or exp alkylating agent/

#51 limit 50 to (human and clinical trial and "therapy (maximizes sensitivity)" and article and adult <18 to 64 years> and "humans only (removes records about animals)")

#52 exp hematopoietic stem cell/ or stem cell transplant*.mp.

#53 limit 52 to (human and clinical trial and "therapy (maximizes sensitivity)" and article and adult <18 to 64 years> and "humans only (removes records about animals)")

#54 marrow ablative chemotherapy.mp.

#55 limit 54 to (human and clinical trial and "therapy (maximizes sensitivity)" and article and adult <18 to 64 years> and "humans only (removes records about animals)")

#56 myeloablative chemotherapy.mp.

#57 limit 56 to (human and clinical trial and "therapy (maximizes sensitivity)" and article and adult <18 to 64 years> and "humans only (removes records about animals)")

**#58 39 or 41 or 43 or 45 or 47 or 49 or 51 or 53 or 55 or 57**

**#59 37 and 58**

**Appendix 3.**

**Search strategy for CENTRAL**

**#1 MeSH descriptor: [Testicular Neoplasms] explode all trees**

**#2 ("testicular cancer"): ti, ab, kw**

**#3 testicular cancer**

**#4 (cancer of the testis): ti, ab, kw**

**#5 cancer of the testis**

**#6 (tumor of the testis): ti, ab, kw**

**#7 tumor of the testis**

**#8 (testicular malignancy): ti, ab, kw**

**#9 testicular malignancy**

**#10 (germ cell tumor): ti, ab, kw**

**#11 germ cell tumor**

**#12 (extragonadal germ cell tumor): ti, ab, kw**

**#13 extragonadal germ cell tumor**

**#14 ("testicular germ-cell tumor"): ti, ab, kw**

**#15 testicular germ cell tumor**

**#16 (mediastinal germ cell tumor): ti, ab, kw**

**#17 mediastinal germ cell tumor**

**#18 (retroperitoneal germ cell tumor): ti, ab, kw**

**#19 retroperitoneal germ cell tumor**

**#20 (intracranial germ cell tumor): ti, ab, kw**

**#21 intracranial germ cell tumor**

**#22 MeSH descriptor: [Neoplasms, Germ Cell and Embryonal] explode all trees**

**#23 (seminoma): ti, ab, kw**

**#24 MeSH descriptor: [Seminoma] explode all trees**

**#25 ("nonseminomatous germ cell tumors"): ti, ab, kw**

**#26 ("yolk sac tumor"): ti, ab, kw**

**#27 ("choriocarcinoma"): ti, ab, kw**

**#28 ("embryonal carcinoma"): ti, ab, kw**

**#29 teratoma**

**#30 #1 or #2 or #4 or #6 or #8 or #10 or # 12 or #14 or #16 or #18 or #20 or #22 or #23 or #24 or #25 or #26 or #27 or #28 or #29 in Trials**

**#31 high dose chemotherapy**

**#32 (high dose chemotherapy): ti, ab, kw**

**#33 bone marrow transplant***

**#34 (bone marrow transplant*): ti, ab, kw**

**#35 MeSH descriptor: [Bone Marrow Transplantation] explode all trees**

**#36 carboplatin**

**#37 (carboplatin): ti, ab, kw**

**#38 MeSH descriptor: [Carboplatin] explode all trees**

**#39 etoposide**

**#40 (etoposide): ti, ab, kw**

**#41 MeSH descriptor: [Etoposide] explode all trees**

**#42 cyclophosphamide**

**#43 (cyclophosphamide): ti, ab, kw**

**#44 MeSH descriptor: [Cyclophosphamide] explode all trees**

**#45 ifosfamide**

**#46 (ifosfamide): ti, ab, kw**

**#47 MeSH descriptor: [Ifosfamide] explode all trees**

**#48 alkylating agents**

**#49 (alkylating agents): ti, ab, kw**

**#50 MeSH descriptor: [Alkylating Agents] explode all trees**

**#51 stem cell transplant***

**#52 (stem cell transplant*): ti, ab, kw**

**#53 MeSH descriptor: [Stem Cell Transplantation] explode all trees**

**#54 marrow ablative chemotherapy**

**#55 (marrow ablative chemotherapy): ti, ab, kw**

**#56 myeloablative chemotherapy**

**#57 (myeloablative chemotherapy): ti, ab, kw**

**#58 #32 #34 or #35 or #37 or #38 or # 40 or #41 or #43 or #44 or #46 or #47 or #49 or #50 or #52 or #53 or #55 or #56**

**#59 #30 and #58 with Publication Year from 1960 to 2023, in Trials with 'Urology', 'Gynaecological, Neuro-oncology and Orphan Cancer', 'Childhood Cancer' in Cochrane Group**

**Appendix 4.**

**Stata commands**

**OS: IT94 trial (confirmatory analysis)**

metan lnHR selnHR, label(namevar =Study) fixed lcols(N lnHR selnHR) eform effect(HR(OS)) nowt forestplot(xlabel(0.65 1 1.5,force) boxopts(mcolor(red)) ciopts(lcolor(black) rcap) nlineopts(lcolor(black))) textsize(130) favours(Favours HDCT # Favours CDCT, fp(1.5)) astext(50) nohet

*********************************************************************************************

**EFS: IT94 trial (exploratory analysis)**

metan lnHR selnHR, label(namevar =Study) fixed lcols(N lnHR selnHR) eform effect(HR(EFS)) nowt forestplot(xlabel(0.55 1 1.15,force) boxopts(mcolor(red)) ciopts(lcolor(black) rcap) nlineopts(lcolor(black))) textsize(130) favours(Favours HDCT # Favours CDCT, fp(1.2)) astext (50) nohet

*********************************************************************************************

**Response Rate (exploratory analysis)**

gen CRctnoevent_exp= N_exp - CRctonlyn_exp

gen CRctnoevent_control= N_control - CRctonlyn_control

gen CRcombnoevent_exp= N_exp - CRctsurgeryn_exp

gen CRcombnoevent_control= N_control - CRctsurgeryn_control

gen CR_exp= CRctonlyn_exp + CRctsurgeryn_exp

gen CR_control= CRctonlyn_control + CRctsurgeryn_control

gen NoCR_exp = N_exp - CR_exp

gen NoCR_control = N_control - CR_control

gen PRTMnegnoevent_exp= N_exp - PRnormaltumormarkersn_exp

gen PRTMnegnoevent_control= N_control - PRnormaltumormarkersn_cont

gen Failuren_exp= PRelevatedtumormarkersn_ex + SDn_exp + PDn_exp

gen Failuren_control= PRelevatedtumormarkersn_co + SDn_control + PDn_control

gen NoFailuren_exp= N_exp - Failuren_exp

gen NoFailuren_control= N_control - Failuren_control

gen ORR_exp = CRctonlyn_exp + CRctsurgeryn_exp + PRnormaltumormarkersn_exp

gen ORR_control = CRctonlyn_control + CRctsurgeryn_control + PRnormaltumormarkersn_cont

gen NoORR_exp = N_exp - ORR_exp

gen NoORR_control = N_control - ORR_control

**Response rate: IT94 trial**

metan ORR_exp NoORR_exp ORR_control NoORR_control, mhaenszel label(namevar =Study) counts npts group1(CDCT) group2(HDCT) effect(RR:ORR) nowt forestplot(xlabel(0.8 1 1.3,force) boxopts(mcolor(red)) ciopts(lcolor(black) rcap) nlineopts(lcolor(black)))textsize(110) favours(Favours CDCT # Favours HDCT, fp(1.3)) astext(50) nohet

metan Failuren_exp NoFailuren_exp Failuren_control NoFailuren_control, mhaenszel nohet nobetween label(namevar =Study) counts npts group1(CDCT) group2(HDCT) effect(RR:Failure) nowt forestplot(xlabel(0.64 1 1.4,force) boxopts(mcolor(red)) ciopts(lcolor(black) rcap) nlineopts(lcolor(black)))textsize(90) favours(Favours HDCT # Favours CDCT, fp(1.5)) astext(60)

**Response rate: all included studies**

metan Failuren_exp NoFailuren_exp Failuren_control NoFailuren_control, mhaenszel label(namevar =Study) counts npts group1(CDCT) group2(HDCT) effect(RR:Failure) nowt forestplot(xlabel(0.2 1 1.6,force) boxopts(mcolor(red)) ciopts(lcolor(black) rcap) nlineopts(lcolor(black)))textsize(100) favours(Favours HDCT # Favours CDCT, fp(2)) astext(50)

metan CR_exp NoCR_exp CR_control NoCR_control, mhaenszel label(namevar =Study) counts npts group1(CDCT) group2(HDCT) effect(RR:CR) nowt forestplot(xlabel(0.5 1 2 9,force) boxopts(mcolor(red)) ciopts(lcolor(black) rcap) nlineopts(lcolor(black)))textsize(100) favours(Favours CDCT # Favours HDCT, fp(2)) astext(45)

*********************************************************************************************

**Acute toxicity: (exploratory analysis)**

gen NoLeukopenia_exp = N_exp - LeukopeniaG3_exp

gen NoLeukopenia_control = N_control - LeukopeniaG3_control

gen NoAnemia_exp = N_exp - AnemiaG3_exp

gen NoAnemia_control = N_control - AnemiaG3_control

gen Neutropenianoevent_exp= N_exp - NeutropeniaG3_exp

gen Neutropenianoevent_control= N_control - NeutropeniaG3_control

gen NoFN_exp= N_exp - FebrileneutropeniaG3_exp

gen NoFN_control= N_control - FebrileneutropeniaG3_control

gen NoThromb_exp= N_exp - ThrombocytopeniaG3_exp

gen NoThromb_control= N_control - ThrombocytopeniaG3_cont

gen NoNV_exp = N_exp - NauseavomitingG3_exp

gen NoNV_control = N_control - NauseavomitingG3_control

gen NoD_exp = N_exp - DiarrhoeaG3_exp

gen NoD_control = N_control - DiarrhoeaG3_control

gen NoM_exp = N_exp - MucositisG3_exp

gen NoM_control = N_control - MucositisG3_control

gen NoDOT_exp = N_exp - Deathduetotoxicity_exp

gen NoDOT_control = N_control - Deathduetotoxicity_control

**Acute hematological toxicity: IT94 trial**

metan NeutropeniaG3_exp Neutropenianoevent_exp NeutropeniaG3_control Neutropenianoevent_control, mhaenszel counts npts group1(CDCT) group2(HDCT) label(namevar =Study) effect(RR:Neutropenia G3+) nowt forestplot(xlabel(0.4 1 1.4,force) boxopts(mcolor(red)) ciopts(lcolor(black) rcap) nlineopts(lcolor(black)))textsize(100) favours(Favours CDCT # Favours HDCT, fp(1.5)) astext(50)

metan FebrileneutropeniaG3_exp NoFN_exp FebrileneutropeniaG3_control NoFN_control, mhaenszel counts npts group1(CDCT) group2(HDCT) label(namevar =Study) effect(RR:Febrile neutropenia G3+) nowt forestplot(xlabel(0.3 0.5 1 1.5 2 2.5,force) boxopts(mcolor(red)) ciopts(lcolor(black) rcap) nlineopts(lcolor(black)))textsize(100) favours(Favours CDCT # Favours HDCT, fp(1.5)) astext(50)

metan ThrombocytopeniaG3_exp NoThromb_exp ThrombocytopeniaG3_cont NoThromb_control, mhaenszel counts npts group1(CDCT) group2(HDCT) label(namevar =Study) effect(RR:Thrombocytopenia G3+) nowt forestplot(xlabel(0.2 0.5 1 1.5 2,force) boxopts(mcolor(red)) ciopts(lcolor(black) rcap) nlineopts(lcolor(black)))textsize(100) favours(Favours CDCT # Favours HDCT, fp(1.8)) astext(50)

**Non hematological toxicity: IT94 trial**

metan NauseavomitingG3_exp NoNV_exp NauseavomitingG3_control NoNV_control, mhaenszel nohet counts label(namevar =Study) effect(RR:Nausea and Vomiting G3+) nowt forestplot(xlabel(0.5 1 2 6,force) boxopts(mcolor(red)) ciopts(lcolor(black) rcap) nlineopts(lcolor(black)))textsize(100) favours(Favours CDCT # Favours HDCT, fp(2)) astext(60)

metan DiarrhoeaG3_exp NoD_exp DiarrhoeaG3_control NoD_control, mhaenszel nohet counts label(namevar =Study) effect(RR:Diarrhoea G3+) nowt forestplot(xlabel(0.5 1 2 10 50,force) boxopts(mcolor(red)) ciopts(lcolor(black) rcap) nlineopts(lcolor(black)))textsize(100) favours(Favours CDCT # Favours HDCT, fp(2)) astext(60)

metan MucositisG3_exp NoM_exp MucositisG3_control NoM_control, mhaenszel nohet counts label(namevar =Study) effect(RR:Mucositis G3+) nowt forestplot(xlabel(0.5 1 5 25 55,force) boxopts(mcolor(red)) ciopts(lcolor(black) rcap) nlineopts(lcolor(black)))textsize(100) title (HDCT vs CDCT: IT94 trial) favours(Favours CDCT # Favours HDCT, fp(5)) astext(60)

metan Deathduetotoxicity_exp NoDOT_exp Deathduetotoxicity_control NoDOT_control, mhaenszel counts label(namevar =Study) effect(RR:DOT) nowt forestplot(xlabel(0.01 0.05 0.5 1 5 20 40,force) boxopts(mcolor(red)) ciopts(lcolor(black) rcap) nlineopts(lcolor(black)))textsize(100) favours(Favours CDCT # Favours HDCT, fp(5)) astext(50)

**Acute hematological toxicity: Mardiak´s study**

metan LeukopeniaG3_exp NoLeukopenia_exp LeukopeniaG3_control NoLeukopenia_control, mhaenszel counts npts group1(CDCT) group2(HDCT) label(namevar =Study) effect(RR:Leukopenia G3+) nowt forestplot(xlabel(0.4 1 1.4,force) boxopts(mcolor(red)) ciopts(lcolor(black) rcap) nlineopts(lcolor(black)))textsize(100) favours(Favours CDCT # Favours HDCT, fp(1.5)) astext(50)

metan AnemiaG3_exp NoAnemia_exp AnemiaG3_control NoAnemia_control, mhaenszel counts npts group1(CDCT) group2(HDCT) label(namevar =Study) effect(RR:Neutropenia G3+) nowt forestplot(xlabel(0.4 1 1.4,force) boxopts(mcolor(red)) ciopts(lcolor(black) rcap) nlineopts(lcolor(black)))textsize(100) favours(Favours CDCT # Favours HDCT, fp(1.5)) astext(50)

**Reference**

1. Pico JL, Rosti G, Kramar A, et al. A randomised trial of high-dose chemotherapy in the salvage treatment of patients failing first-line platinum chemotherapy for advanced germ cell tumours. *Ann Oncol.* 2005;16(7):1152-1159.

2. Lorch A, Kollmannsberger C, Hartmann JT, et al. Single versus sequential high-dose chemotherapy in patients with relapsed or refractory germ cell tumors: a prospective randomized multicenter trial of the German Testicular Cancer Study Group. *J Clin Oncol.* 2007;25(19):2778-2784.

3. Ongoing Clinical Trials in Testicular Cancer: The TIGER Trial. *Oncol Res Treat.* 2016;39(9):553-556.

4. Al-Ezzi EM, Zahralliyali A, Hansen AR, et al. The Use of Salvage Chemotherapy for Patients with Relapsed Testicular Germ Cell Tumor (GCT) in Canada: A National Survey. *Current Oncology.* 2023;30(7):6166-6176.

5. Husnain M, Riaz IB, Kamal MU, et al. High Dose Chemotherapy with Autologous Stem Cell Transplant in Treatment of Germ Cell Tumors: A Systematic Review and Meta-Analysis. *Blood.* 2016;128(22):5829-5829.

6. Bin Riaz I, Umar M, Zahid U, et al. Role of one, two and three doses of high-dose chemotherapy with autologous transplantation in the treatment of high-risk or relapsed testicular cancer: a systematic review. *Bone Marrow Transplant.* 2018;53(10):1242-1254.

7. Page MJ, McKenzie JE, Bossuyt PM, et al. The PRISMA 2020 statement: An updated guideline for reporting systematic reviews.

8. Moher D, Shamseer L, Clarke M, et al. Preferred Reporting Items for Systematic Review and Meta-Analysis Protocols (PRISMA-P) 2015 statement. Syst Rev. 2015;4(1):1.

9. Petrelli F, Coinu A, Rosti G, Pedrazzoli P, Barni S. Salvage treatment for testicular cancer with standard- or high-dose chemotherapy: a systematic review of 59 studies. *Med Oncol.* 2017;34(8):133.

10. Chovanec M, Adra N, Abu Zaid M, Abonour R, Einhorn L. High-dose chemotherapy for relapsed testicular germ cell tumours. *Nature Reviews Urology.* 2022.

11. Tierney JF, Stewart LA, Ghersi D, Burdett S, Sydes MR. Practical methods for incorporating summary time-to-event data into meta-analysis. *Trials.* 2007;8(1):16.

12. Sterne JAC, Savović J, Page MJ, et al. RoB 2: a revised tool for assessing risk of bias in randomised trials. BMJ 2019; 366: l4898.

13. Sterne JAC, Hernán MA, Reeves BC, et al. ROBINS-I: a tool for assessing risk of bias in non-randomized studies of interventions. BMJ 2016; 355; i4919; doi: 10.1136/bmj.i4919

14. Deeks JJ, Higgins JPT, Altman DG (editors). Chapter 10: Analysing data and undertaking meta-analyses. In: Higgins JPT, Thomas J, Chandler J, Cumpston M, Li T, Page MJ, Welch VA (editors). Cochrane Handbook for Systematic Reviews of Interventions version 6.4 (updated August 2023). Cochrane, 2023. Available from <www.training.cochrane.org/handbook>.

15. Gagnier JJ, Moher D, Boon H, Beyene J, Bombardier C. Investigating clinical heterogeneity in systematic reviews: a methodologic review of guidance in the literature. *BMC Med Res Methodol.* 2012;12:111.

16. Gagnier JJ, Morgenstern H, Altman DG, et al. Consensus-based recommendations for investigating clinical heterogeneity in systematic reviews. *BMC Med Res Methodol.* 2013;13:106.

17. Proschan MA, Waclawiw MA. Practical guidelines for multiplicity adjustment in clinical trials. *Control Clin Trials.* 2000;21(6):527-539.

18. (McMaster University and Evidence Prime., 2022)

19. Schünemann HJ, Higgins JPT, Vist GE, et al. Chapter 14: Completing ‘Summary of findings’ tables and grading the certainty of the evidence. In: Higgins JPT, Thomas J, Chandler J, Cumpston M, Li T, Page MJ, Welch VA (editors). Cochrane Handbook for Systematic Reviews of Interventions version 6.4 (updated August 2023). Cochrane, 2023. Available from <www.training.cochrane.org/handbook>.

20. Mardiak J, Fuchsberger P, Lakota J, et al. Sequential intermediate high-dose therapy with etoposide, ifosfamide and cisplatin for patients with germ cell tumors. *Neoplasma.* 2000;47(4):239-243.

21. Faure-Conter C, Orbach D, Cropet C, et al. Salvage therapy for refractory or recurrent pediatric germ cell tumors: the French SFCE experience. *Pediatr Blood Cancer.* 2014;61(2):253-259.

22. ACTRN12618001236280. Standard-Dose Combination Chemotherapy or High-Dose Combination Chemotherapy and Stem Cell Transplant in Treating Patients With Relapsed or Refractory Germ Cell Tumors. In. Alliance for Clinical Trials in Oncology Y, Australian, New Zealand U, Prostate Cancer Trials A, trans2018.

23. McGuinness LA, Higgins JPT. Risk-of-bias VISualization (robvis): An R package and Shiny web app for visualizing risk-of-bias assessments. *Research Synthesis Methods.* 2020;n/a(n/a).

24. Buckner CD, Clift RA, Fefer A, et al. High-dose cyclophosphamide (NSC-26271) for the treatment of metastatic testicular neoplasms. *Cancer Chemother Rep.* 1974;58(5 Pt 1):709-714.

25. DeWys WD, Begg C, Slayton R, Hahn RG, Brodsky I. Chemotherapy for advanced germinal cell neoplasms: preliminary report of an Eastern Cooperative Oncology Group Study. *Cancer treatment reports.* 1979;63(9‐10):1675‐1680.

26. Blijham G, Spitzer G, Litam J, et al. The treatment of advanced testicular carcinoma with high dose chemotherapy and autologous marrow support. *Eur J Cancer (1965).* 1981;17(4):433-441.

27. Stoter G, Sleyfer DT, ten Bokkel Huinink WW, et al. High-dose versus low-dose vinblastine in cisplatin-vinblastine-bleomycin combination chemotherapy of non-seminomatous testicular cancer: a randomized study of the EORTC Genitourinary Tract Cancer Cooperative Group. *J Clin Oncol.* 1986;4(8):1199-1206.

28. Ozols RF. Treatment of poor prognosis germ cell tumours with high dose cisplatin regimens. *International journal of andrology.* 1987;10(1):291‐300.

29. Ghosn M, Droz JP, Theodore C, et al. Salvage chemotherapy in refractory germ cell tumors with etoposide (VP-16) plus ifosfamide plus high-dose cisplatin. A VIhP regimen. *Cancer.* 1988;62(1):24-27.

30. Mulder PO, de Vries EG, Koops HS, et al. Chemotherapy with maximally tolerable doses of VP 16-213 and cyclophosphamide followed by autologous bone marrow transplantation for the treatment of relapsed or refractory germ cell tumors. *Eur J Cancer Clin Oncol.* 1988;24(4):675-679.

31. Ozols RF, Ihde DC, Linehan WM, Jacob J, Ostchega Y, Young RC. A randomized trial of standard chemotherapy v a high-dose chemotherapy regimen in the treatment of poor prognosis nonseminomatous germ-cell tumors. *Journal of Clinical Oncology.* 1988;6(6):1031-1040.

32. Nichols CR, Tricot G, Williams SD, et al. Dose-intensive chemotherapy in refractory germ cell cancer--a phase I/II trial of high-dose carboplatin and etoposide with autologous bone marrow transplantation. *J Clin Oncol.* 1989;7(7):932-939.

33. Broun ER, Nichols CR, Tricot G, Loehrer PJ, Williams SD, Einhorn LH. High dose carboplatin/VP-16 plus ifosfamide with autologous bone marrow support in the treatment of refractory germ cell tumors. *Bone Marrow Transplant.* 1991;7(1):53-56.

34. Rosti G, Albertazzi L, Salvioni R, et al. High dose chemotherapy with carboplatin, VP 16 +/- ifosfamide in germ cell tumors: the Italian experience. *Bone Marrow Transplant.* 1991;7 Suppl 2:94.

35. Elias AD, Ayash LJ, Eder JP, et al. A phase I study of high-dose ifosfamide and escalating doses of carboplatin with autologous bone marrow support. *J Clin Oncol.* 1991;9(2):320-327.

36. Broun ER, Nichols CR, Kneebone P, et al. Long-term outcome of patients with relapsed and refractory germ cell tumors treated with high-dose chemotherapy and autologous bone marrow rescue. *Ann Intern Med.* 1992;117(2):124-128.

37. Rosti G, Albertazzi L, Salvioni R, et al. High-dose chemotherapy supported with autologous bone marrow transplantation (ABMT) in germ cell tumors: a phase two study. *Ann Oncol.* 1992;3(10):809-812.

38. Rodenhuis S, Vlasveld LT, Dubbelman R, et al. Feasibility study of high-dose carboplatin and etoposide in the salvage treatment of testicular cancer. *Annals of Oncology.* 1992;3(6):463-467.

39. Santana VM, Schell MJ, Williams R, et al. Escalating sequential high-dose carboplatin and etoposide with autologous marrow support in children with relapsed solid tumors. *Bone Marrow Transplant.* 1992;10(5):457-462.

40. Droz JP, Pico JL, Ghosn M, et al. A phase II trial of early intensive chemotherapy with autologous bone marrow transplantation in the treatment of poor prognosis non seminomatous germ cell tumors. *Bulletin du Cancer.* 1992;79(5):497‐507.

41. Motzer RJ, Gulati SC, Crown JP, et al. High-dose chemotherapy and autologous bone marrow rescue for patients with refractory germ cell tumors. Early intervention is better tolerated. *Cancer.* 1992;69(2):550-556.

42. Nichols CR, Andersen J, Lazarus HM, et al. High-dose carboplatin and etoposide with autologous bone marrow transplantation in refractory germ cell cancer: an Eastern Cooperative Oncology Group protocol. *J Clin Oncol.* 1992;10(4):558-563.

43. Guimaraes A, Camba L, Hall G, et al. High dose chemotherapy followed by autologous bone marrow transplant for refractory germ cell tumours. *Leuk Lymphoma.* 1992;7 Suppl:65-68.

44. Rosti G, Albertazzi L, Tienghi A, et al. An Italian experience of high-dose chemotherapy and autologous bone marrow transplantation (ABMT) in germ cell tumours. Suggestions for future direction. *Leuk Lymphoma.* 1992;7 Suppl:59-63.

45. Barnett MJ, Coppin CM, Murray N, et al. High-dose chemotherapy and autologous bone marrow transplantation for patients with poor prognosis nonseminomatous germ cell tumours. *Br J Cancer.* 1993;68(3):594-598.

46. Motzer RJ, Gulati SC, Tong WP, et al. Phase I trial with pharmacokinetic analyses of high-dose carboplatin, etoposide, and cyclophosphamide with autologous bone marrow transplantation in patients with refractory germ cell tumors. *Cancer Res.* 1993;53(16):3730-3735.

47. Chevreau C, Droz JP, Pico JL, et al. Early intensified chemotherapy with autologous bone marrow transplantation in first line treatment of poor risk non-seminomatous germ cell tumours. Preliminary results of a French randomized trial. *Eur Urol.* 1993;23(1):213-217; discussion 218.

48. Broun ER, Nichols CR, Turns M, et al. Early salvage therapy for germ cell cancer using high dose chemotherapy with autologous bone marrow support. *Cancer.* 1994;73(6):1716-1720.

49. Siegert W, Beyer J, Strohscheer I, et al. High-dose treatment with carboplatin, etoposide, and ifosfamide followed by autologous stem-cell transplantation in relapsed or refractory germ cell cancer: a phase I/II study. The German Testicular Cancer Cooperative Study Group. *J Clin Oncol.* 1994;12(6):1223-1231.

50. Lotz JP, André T, Donsimoni R, et al. High dose chemotherapy with ifosfamide, carboplatin, and etoposide combined with autologous bone marrow transplantation for the treatment of poor-prognosis germ cell tumors and metastatic trophoblastic disease in adults. *Cancer.* 1995;75(3):874-885.

51. Rodenhuis S, van der Wall E, ten Bokkel Huinink WW, Schornagel JH, Richel DJ, Vlasveld LT. Pilot study of a high-dose carboplatin-based salvage strategy for relapsing or refractory germ cell cancer. *Cancer Invest.* 1995;13(4):355-362.

52. Lampe H, Dearnaley DP, Price A, et al. High-dose carboplatin and etoposide for salvage chemotherapy of germ cell tumours. *Eur J Cancer.* 1995;31a(5):717-723.

53. Beyer J, Schwella N, Zingsem J, et al. Hematopoietic rescue after high-dose chemotherapy using autologous peripheral-blood progenitor cells or bone marrow: a randomized comparison. *J Clin Oncol.* 1995;13(6):1328-1335.

54. Takeda M, Sakamaki S, Watanabe N. Studies on clinical significance of high-dose chemotherapy with peripheral blood stem cell transplantation for patients with solid tumors and malignant lymphoma. *Sapporo Medical Journal.* 1995;64(1-2):1-12.

55. Ladenstein R, Gadner H, Hartmann O, Pico J, Biron P, Thierry P. [The European experience with megadose therapy and autologous bone marrow transplantation in solid tumors with poor prognosis Ewing sarcoma, germ cell tumors and brain tumors)]. *Wien Med Wochenschr.* 1995;145(2-3):55-57.

56. Van Warmerdam LJC, Rodenhuis S, Van Der Wall E, Maes RAA, Beijnen JH. Pharmacokinetics and pharmacodynamics of carboplatin administered in a high-dose combination regimen with thiotepa, cyclophosphamide and peripheral stem cell support. *British Journal of Cancer.* 1996;73(8):979-984.

57. Farhat F, Culine S, Théodore C, Békradda M, Terrier-Lacombe MJ, Droz JP. Cisplatin and ifosfamide with either vinblastine or etoposide as salvage therapy for refractory or relapsing germ cell tumor patients: the Institut Gustave Roussy experience. *Cancer.* 1996;77(6):1193-1197.

58. Motzer RJ, Mazumdar M, Bosl GJ, Bajorin DF, Amsterdam A, Vlamis V. High-dose carboplatin, etoposide, and cyclophosphamide for patients with refractory germ cell tumors: treatment results and prognostic factors for survival and toxicity. *J Clin Oncol.* 1996;14(4):1098-1105.

59. Margolin K, Doroshow JH, Ahn C, et al. Treatment of germ cell cancer with two cycles of high-dose ifosfamide, carboplatin, and etoposide with autologous stem-cell support. *Journal of Clinical Oncology.* 1996;14(10):2631-2637.

60. Fondazione Irccs Istituto Nazionale dei Tumori M. Conventional Dose Versus High Dose Sequential Chemotherapy for Poor Prognosis Germ Cell Tumors. In: <https://ClinicalTrials.gov/show/NCT02161692>; 1996.

61. Beyer J, Kingreen D, Krause M, et al. Long-term survival of patients with recurrent or refractory germ cell tumors after high dose chemotherapy. *Cancer.* 1997;79(1):161-168.

62. Broun ER, Nichols CR, Gize G, et al. Tandem high dose chemotherapy with autologous bone marrow transplantation for initial relapse of testicular germ cell cancer. *Cancer.* 1997;79(8):1605-1610.

63. Graham ML, Herndon IJE, Casey JR, et al. High-dose chemotherapy with autologous stem-cell rescue in patients with recurrent and high-risk pediatric brain tumors. *Journal of Clinical Oncology.* 1997;15(5):1814-1823.

64. Motzer RJ, Mazumdar M, Bajorin DF, Bosl GJ, Lyn P, Vlamis V. High-dose carboplatin, etoposide, and cyclophosphamide with autologous bone marrow transplantation in first-line therapy for patients with poor- risk germ cell tumors. *Journal of Clinical Oncology.* 1997;15(7):2546-2552.

65. Memorial Sloan Kettering Cancer C, National Cancer I. High-Dose Thiotepa Plus Peripheral Stem Cell Transplantation in Treating Patients With Refractory Solid Tumors. In: <https://ClinicalTrials.gov/show/NCT00003173>; 1997.

66. Mandanas RA, Saez RA, Epstein RB, Confer DL, Selby GB. Long-term results of autologous marrow transplantation for relapsed or refractory male or female germ cell tumors. *Bone Marrow Transplant.* 1998;21(6):569-576.

67. Papadopoulos KP, Garvin JH, Fetell M, et al. High-dose thiotepa and etoposide-based regimens with autologous hematopoietic support for high-risk or recurrent CNS tumors in children and adults. *Bone Marrow Transplantation.* 1998;22(7):661-667.

68. Kanfer EJ, McGuigan D, Samson D, et al. High-dose etoposide with granulocyte colony-stimulating factor for mobilization of peripheral blood progenitor cells: efficacy and toxicity at three dose levels. *Br J Cancer.* 1998;78(7):928-932.

69. Lyttelton MP, Newlands ES, Giles C, et al. High-dose therapy including carboplatin adjusted for renal function in patients with relapsed or refractory germ cell tumour: outcome and prognostic factors. *Br J Cancer.* 1998;77(10):1672-1676.

70. Rodenhuis S, de Wit R, de Mulder PH, et al. A multi-center prospective phase II study of high-dose chemotherapy in germ-cell cancer patients relapsing from complete remission. *Ann Oncol.* 1999;10(12):1467-1473.

71. Shamash J, Oliver RTD, Ong J, et al. Sixty percent salvage rate for germ-cell tumours using sequential m-BOP, surgery and ifosfamide-based chemotherapy. *Annals of Oncology.* 1999;10(6):685-692.

72. Hara I, Yamada Y, Miyake H, et al. Clinical outcome of high-dose chemotherapy combined with peripheral blood stem cell transplantation for male germ cell tumors. *Anticancer Drugs.* 1999;10(8):711-718.

73. Adjuvant Chemotherapy for Muscle-invasive Bladder Cancer: A Systematic Review and Meta-analysis of Individual Participant Data from Randomised Controlled Trials. *Eur Urol.* 2022;81(1):50-61.

74. Nakagawa S, Miki T, Akaza H, et al. [High-dose chemotherapy with peripheral blood stem cell autotransplantation for patients with poor-risk testicular germ cell tumors--pilot study of the Japan Blood Cell Transplantation Study Group]. *Hinyokika Kiyo.* 1999;45(11):805-809.

75. Bokemeyer C, Kollmannsberger C, Meisner C, et al. First-Line High-Dose Chemotherapy Compared With Standard-Dose PEB/VIP Chemotherapy in Patients With Advanced Germ Cell Tumors: A Multivariate and Matched-Pair Analysis. *Journal of Clinical Oncology.* 1999;17(11):3450-3456.

76. Fosså SD, Kaye SB, Mead GM, et al. Filgrastim during combination chemotherapy of patients with poor-prognosis metastatic germ cell malignancy. European Organization for Research and Treatment of Cancer, Genito-Urinary Group, and the Medical Research Council Testicular Cancer Working Party, Cambridge, United Kingdom. *J Clin Oncol.* 1998;16(2):716-724.

77. Nct, Temple University N, Fox Chase Cancer C. Combination Chemotherapy Followed by Bone Marrow or Stem Cell Transplantation in Treating Patients With Relapsed or Refractory Germ Cell Tumors. In:1999.

78. Nct, Memorial Sloan Kettering Cancer Center N, National Cancer Institute Yes 23/05/ <https://clinicaltrials.gov/ct2/show/results> NCT. Combination Chemotherapy Plus Peripheral Stem Cell Transplantation in Treating Patients With Germ Cell Tumors. In:1999.

79. Nct, Children's Oncology Group N, National Cancer I. Combination Chemotherapy Followed by Bone Marrow and/or Peripheral Stem Cell Transplantation in Treating Patients With Recurrent Medulloblastoma or CNS Germ Cell Tumors. In:1999.

80. Nct, Memorial Sloan Kettering Cancer Center N, National Cancer I, et al. Combination Chemotherapy With or Without Bone Marrow or Stem Cell Transplantation in Treating Men With Untreated Germ Cell Tumors. In:1999.

81. Nct, City of Hope Medical Center N, National Cancer Institute Yes 04/01/ <https://clinicaltrials.gov/ct2/show/results> NCT. Combination Chemotherapy Plus Peripheral Stem Cell Transplantation in Treating Patients With Relapsed Germ Cell Cancer. In:1999.

82. Nct, Wake Forest University Health Sciences N, National Cancer I. Carboplatin, Etoposide, Cyclophosphamide, and Autologous Bone Marrow Transplantation in Patients With Relapsed or Refractory Cancer. In:1999.

83. Nct, Memorial Sloan Kettering Cancer Center N, National Cancer I. High-Dose Thiotepa Plus Peripheral Stem Cell Transplantation in Treating Patients With Refractory Solid Tumors. In:1999.

84. Nct, Children's Oncology Group Y, National Cancer I. Combination Chemotherapy Plus Amifostine in Treating Children With Malignant Germ Cell Tumors. In:1999.

85. Shamash J, O'Doherty CA, Oliver RT, et al. Should high-dose chemotherapy be used to consolidate second or third line treatment in relapsing germ cell tumours? *Acta Oncol.* 2000;39(7):857-863.

86. Kollmannsberger C, Nichols C, Bamberg M, et al. First-line high-dose chemotherapy +/- radiation therapy in patients with metastatic germ-cell cancer and brain metastases. *Ann Oncol.* 2000;11(5):553-559.

87. Decatris MP, Wilkinson PM, Welch RS, Metzner M, Morgenstern GR, Dougall M. High-dose chemotherapy and autologous haematopoietic support in poor risk non-seminomatous germ-cell tumours: An effective first-line therapy with minimal toxicity. *Annals of Oncology.* 2000;11(4):427-434.

88. Miyazaki J, Miyanaga N, Kawai K, Shimazui T, Takeshima H, Akaza H. High-dose chemotherapy with peripheral blood stem cell transplantation for advanced testicular cancer. *Int J Urol.* 2000;7(7):258-262.

89. Bhatia S, Abonour R, Porcu P, et al. High-dose chemotherapy as initial salvage chemotherapy in patients with relapsed testicular cancer. *J Clin Oncol.* 2000;18(19):3346-3351.

90. Motzer RJ, Mazumdar M, Sheinfeld J, et al. Sequential dose-intensive paclitaxel, ifosfamide, carboplatin, and etoposide salvage therapy for germ cell tumor patients. *J Clin Oncol.* 2000;18(6):1173-1180.

91. Pierluigi P, Sumeet B, Matt S, Lawrence HE. Results of Treatment After Relapse From High-Dose Chemotherapy in Germ Cell Tumors. *Journal of Clinical Oncology.* 2000;18(6):1181-1186.

92. Nct, Duke University Y, National Cancer I. Temozolomide Plus Peripheral Stem Cell Transplantation in Treating Children With Newly Diagnosed Malignant Glioma or Recurrent CNS or Other Solid Tumors. In:2000.

93. Rick O, Beyer J, Schwella N, Schubart H, Schleicher J, Siegert W. Assessment of amifostine as protection from chemotherapy-induced toxicities after conventional-dose and high-dose chemotherapy in patients with germ cell tumor. *Annals of Oncology.* 2001;12(8):1151-1155.

94. Ayash LJ, Clarke M, Silver SM, et al. Double dose-intensive chemotherapy with autologous stem cell support for relapsed and refractory testicular cancer: the University of Michigan experience and literature review. *Bone Marrow Transplant.* 2001;27(9):939-947.

95. Kohda K, Sakamaki S, Matsunaga T, et al. Long-term survival and late-onset complications of cancer patients treated with high-dose chemotherapy followed by autologous peripheral blood stem cell transplantation. *Int J Hematol.* 2001;73(2):251-257.

96. Rick O, Bokemeyer C, Beyer J, et al. Salvage treatment with paclitaxel, ifosfamide, and cisplatin plus high-dose carboplatin, etoposide, and thiotepa followed by autologous stem-cell rescue in patients with relapsed or refractory germ cell cancer. *J Clin Oncol.* 2001;19(1):81-88.

97. Nct, Sidney Kimmel Comprehensive Cancer Center at Johns Hopkins N, Amgen, Baxter Healthcare C, Nexell Therapeutics I. Peripheral Stem Cell Transplantation Plus Chemotherapy in Treating Patients With Malignant Solid Tumors. In:2001.

98. Nct, Children's Hospital Los Angeles N, National Cancer I. Chemotherapy, Surgery, Radiation Therapy and Bone Marrow or Peripheral Stem Cell Transplantation in Treating Patients With Primary CNS Germ Cell Tumors. In:2001.

99. Rick O, Beyer J, Schwella N, Siegert W. Influence of amifostine on reconstitution of lymphocyte subpopulations after conventional- and high-dose chemotherapy in patients with germ cell tumor. *Annals of Hematology.* 2002;81(12):717-722.

100. Beyer J, Stenning S, Gerl A, Fossa S, Siegert W. High-dose versus conventional-dose chemotherapy asfirst-salvage treatment in patients with non-seminomatousgerm-cell tumors: a matched-pair analysis. *Annals of Oncology.* 2002;13(4):599-605.

101. Rick O, Siegert W, Schwella N, Dubiel M, Krusch A, Beyer J. High-dose chemotherapy as salvage treatment for seminoma. *Bone Marrow Transplantation.* 2002;30(3):157-160.

102. Bokemeyer C, Kollmannsberger C, Oechsle K, et al. Early prediction of treatment response to high-dose salvage chemotherapy in patients with relapsed germ cell cancer using [(18)F]FDG PET. *Br J Cancer.* 2002;86(4):506-511.

103. Rosti G, De Giorgi U, Salvioni R, et al. Salvage high-dose chemotherapy in patients with germ cell tumors: an Italian experience with 84 patients. *Cancer.* 2002;95(2):309-315.

104. Nct, Children's Oncology Group Y, National Cancer Institute Yes 27/02/ <https://clinicaltrials.gov/ct2/show/results> NCT. Neoadjuvant Chemotherapy With or Without Second-Look Surgery Followed by Radiation Therapy With or Without Peripheral Stem Cell Transplantation in Treating Patients With Intracranial Germ Cell Tumors. In:2002.

105. Bokemeyer C, Schleucher N, Metzner B, et al. First-line sequential high-dose VIP chemotherapy with autologous transplantation for patients with primary mediastinal nonseminomatous germ cell tumours: a prospective trial. *Br J Cancer.* 2003;89(1):29-35.

106. Schmoll H-J, Kollmannsberger C, Metzner B, et al. Long-Term Results of First-Line Sequential High-Dose Etoposide, Ifosfamide, and Cisplatin Chemotherapy Plus Autologous Stem Cell Support for Patients With Advanced Metastatic Germ Cell Cancer: An Extended Phase I/II Study of the German Testicular Cancer Study Group. *Journal of Clinical Oncology.* 2003;21(22):4083-4091.

107. Billmire D, Vinocur C, Rescorla F, et al. Malignant retroperitoneal and abdominal germ cell tumors: an intergroup study. *J Pediatr Surg.* 2003;38(3):315-318; discussion 315.

108. Nct, Roswell Park Cancer Institute N. High-Dose Chemotherapy, Total-Body Irradiation, and Autologous Stem Cell Transplantation or Bone Marrow Transplantation in Treating Patients With Hematologic Cancer or Solid Tumors. In:2003.

109. De Giorgi U, Rosti G, Papiani G, Marangolo M. Long-term follow-up of patients with poor prognosis germ cell tumor treated with early high-dose chemotherapy with hematopoietic progenitor cell support: a single-center experience. *Bone Marrow Transplantation.* 2004;33(6):639-643.

110. Rosti G, De Giorgi U, Wandt H, et al. First-line high-dose chemotherapy for patients with poor prognosis extragonadal germ cell tumors: the experience of the European Bone Marrow Transplantation (EBMT) Solid Tumors Working Party. *Bone Marrow Transplantation.* 2004;34(12):1033-1037.

111. McNeish IA, Kanfer EJ, Haynes R, et al. Paclitaxel-containing high-dose chemotherapy for relapsed or refractory testicular germ cell tumours. *Br J Cancer.* 2004;90(6):1169-1175.

112. Cushing B, Giller R, Cullen JW, et al. Randomized comparison of combination chemotherapy with etoposide, bleomycin, and either high-dose or standard-dose cisplatin in children and adolescents with high-risk malignant germ cell tumors: a pediatric intergroup study--Pediatric Oncology Group 9049. *J Clin Oncol.* 2004;22(13):2691-2700.

113. Hartmann JT, Rick O, Thomas M, et al. The role of paclitaxel in the first-line treatment of patients with ‘poor prognosis’ germ cell tumor (GCT) undergoing sequential high dose chemotherapy. *Journal of Clinical Oncology.* 2004;22(14_suppl):4633-4633.

114. Shakeel M, Sharon G, Ira JD, et al. Thiotepa-Based High-Dose Chemotherapy With Autologous Stem-Cell Rescue in Patients With Recurrent or Progressive CNS Germ Cell Tumors. *Journal of Clinical Oncology.* 2004;22(10):1934-1943.

115. Assistance Publique - Hôpitaux de P, Ministry of Health F, Amgen, Baxter Healthcare C. High-dose Chemotherapy With Autologous Stem Cell Transplantation in Poor Prognosis Germ-cell Tumors: TAXIF II. In: <https://ClinicalTrials.gov/show/NCT00231582>; 2004.

116. Lotz JP, Bui B, Gomez F, et al. Sequential high-dose chemotherapy protocol for relapsed poor prognosis germ cell tumors combining two mobilization and cytoreductive treatments followed by three high-dose chemotherapy regimens supported by autologous stem cell transplantation. Results of the phase II multicentric TAXIF trial. *Annals of Oncology.* 2005;16(3):411-418.

117. Margolin KA, Doroshow JH, Frankel P, et al. Paclitaxel-based high-dose chemotherapy with autologous stem cell rescue for relapsed germ cell cancer. *Biol Blood Marrow Transplant.* 2005;11(11):903-911.

118. Nieto Y, Shpall EJ, Bearman SI, et al. Phase I and pharmacokinetic study of docetaxel combined with melphalan and carboplatin, with autologous hematopoietic progenitor cell support, in patients with advanced refractory malignancies. *Biology of Blood and Marrow Transplantation.* 2005;11(4):297-306.

119. Jordan K, Kegel T, Mueller LP, Kinitz I, Schmoll HJ. Feasibility of a combination of high dose chemotherapy ifosfamide, carboplatin, etoposide (ICE) plus stem cells and bevacizumab in refractory sarcoma and germ cell tumors. *Journal of Clinical Oncology.* 2005;23(16_suppl):9070-9070.

120. Banna GL, De Giorgi U, Ferrari B, et al. Is high-dose chemotherapy after primary chemotherapy a therapeutic option for patients with primary mediastinal nonseminomatous germ cell tumor? *Biol Blood Marrow Transplant.* 2006;12(10):1085-1091.

121. Müller AMS, Ihorst G, Waller CF, Dölken G, Finke J, Engelhardt M. Intensive Chemotherapy with Autologous Peripheral Blood Stem Cell Transplantation During a 10-Year Period in 64 Patients with Germ Cell Tumor. *Biology of Blood and Marrow Transplantation.* 2006;12(3):355-365.

122. Fraser CJ, Weigel BJ, Perentesis JP, et al. Autologous stem cell transplantation for high-risk Ewing's sarcoma and other pediatric solid tumors. *Bone Marrow Transplantation.* 2006;37(2):175-181.

123. Bajorin DF, Nichols CR, Margolin KA, et al. Phase III trial of conventional-dose chemotherapy alone or with high-dose chemotherapy for metastatic germ cell tumors (GCT) patients (PTS): A cooperative group trial by Memorial Sloan-Kettering Cancer Center, ECOG, SWOG, and CALGB. *Journal of Clinical Oncology.* 2006;24(18_suppl):4510-4510.

124. Droz JP, Kramar A, Biron P, et al. Failure of high-dose cyclophosphamide and etoposide combined with double-dose cisplatin and bone marrow support in patients with high-volume metastatic nonseminomatous germ-cell tumours: mature results of a randomised trial. *Eur Urol.* 2007;51(3):739-746; discussion 747.

125. Miki T, Mizutani Y, Akaza H, et al. Long-term results of first-line sequential high-dose carboplatin, etoposide and ifosfamide chemotherapy with peripheral blood stem cell support for patients with advanced testicular germ cell tumor. *Int J Urol.* 2007;14(1):54-59.

126. Hartmann JT, Gauler T, Metzner B, et al. Phase I/II study of sequential dose-intensified ifosfamide, cisplatin, and etoposide plus paclitaxel as induction chemotherapy for poor prognosis germ cell tumors by the German Testicular Cancer Study Group. *J Clin Oncol.* 2007;25(36):5742-5747.

127. Kondagunta GV, Bacik J, Sheinfeld J, et al. Paclitaxel plus Ifosfamide followed by high-dose carboplatin plus etoposide in previously treated germ cell tumors. *J Clin Oncol.* 2007;25(1):85-90.

128. Motzer RJ, Nichols CJ, Margolin KA, et al. Phase III randomized trial of conventional-dose chemotherapy with or without high-dose chemotherapy and autologous hematopoietic stem-cell rescue as first-line treatment for patients with poor-prognosis metastatic germ cell tumors. *J Clin Oncol.* 2007;25(3):247-256.

129. Lawrence HE, Mary JB, Beth J, Stephen DW. Phase II Study of Paclitaxel Plus Gemcitabine Salvage Chemotherapy for Germ Cell Tumors After Progression Following High-Dose Chemotherapy With Tandem Transplant. *Journal of Clinical Oncology.* 2007;25(5):513-516.

130. EUCTR2006-006004-11-DE. Phase II – Studie P-ICE Hochdosischemotherapie mit Paclitaxel, Ifosfamid,Carboplatin und Etoposid mit autologem Stammzellsupport bei männlichen Patienten mit refraktären oder rezidivierten Keimzelltumoren. In. Martin-Luther-Universität Halle-Wittenberg vddKY, trans2007.

131. Nct, Memorial Sloan Kettering Cancer Center N, National Cancer Institute Yes 18/05/ <https://clinicaltrials.gov/ct2/show/results> NCT. Paclitaxel, Ifosfamide, and Carboplatin Followed By Autologous Stem Cell Transplant in Treating Patients With Germ Cell Tumors That Did Not Respond to Cisplatin. In:2007.

132. Nct, Masonic Cancer Center UoMN. Autologous Peripheral Blood Stem Cell Transplant for Germ Cell Tumors. In:2007.

133. Nct, Children's C, Leukaemia Group N. Treatment Outcome and Quality of Life in Patients With Pediatric Extra-Cranial Germ Cell Tumors Previously Treated on Clinical Trial CCLG-GC-1979-01 or CCLG-GC-1989-01. In:2007.

134. Nct, Children's Oncology Group Y, National Cancer Institute Yes 20/03/ <https://clinicaltrials.gov/ct2/show/results> NCT. Combination Chemotherapy in Treating Young Patients With Recurrent or Resistant Malignant Germ Cell Tumors. In:2007.

135. Nct, Roswell Park Cancer Institute N, National Cancer I. High-Dose Chemotherapy With or Without Total-Body Irradiation Followed by Autologous Stem Cell Transplant in Treating Patients With Hematologic Cancer or Solid Tumors. In:2007.

136. Nct, University of Southampton N, University Hospital Southampton NHSFT. Gemcitabine, Paclitaxel, Ifosfamide, and Cisplatin in Treating Patients With Progressive or Relapsed Metastatic Germ Cell Tumors. In:2007.

137. Laughton SJ, Merchant TE, Sklar CA, et al. Endocrine outcomes for children with embryonal brain tumors after risk-adapted craniospinal and conformal primary-site irradiation and high-dose chemotherapy with stem-cell rescue on the SJMB-96 trial. *J Clin Oncol.* 2008;26(7):1112-1118.

138. Oechsle K, Lorch A, Honecker FU, et al. Patterns of relapse after primary or salvage high-dose chemotherapy in patients with advanced nonseminomatous germ cell tumors. *Journal of Clinical Oncology.* 2008;26(15_suppl):16012-16012.

139. Ozkaynak MF, Sahdev I, Gross TG, et al. A pilot study of addition of amifostine to melphalan, carboplatin, etoposide, and cyclophosphamide with autologous hematopoietic stem cell transplantation in pediatric solid tumors - A pediatric blood and marrow transplant consortium study. *Journal of Pediatric Hematology/Oncology.* 2008;30(3):204-209.

140. Agarwal R, Dvorak CC, Stockerl-Goldstein KE, Johnston L, Srinivas S. High-dose chemotherapy followed by stem cell rescue for high-risk germ cell tumors: the Stanford experience. *Bone Marrow Transplantation.* 2009;43(7):547-552.

141. Beyer J, Hackenthal M, Lorch A, et al. High-dose chemotherapy (HDCT) as second salvage treatment in patients with multiple relapsed or refractory germ cell tumors. *Journal of Clinical Oncology.* 2009;27(15_suppl):5082-5082.

142. Lotz J, Selle F, Fizazi K, et al. A phase II trial of high-dose chemotherapy (HDCT) supported by haematopoietic stem cell transplantation (HSCT) in patients (pts) with disseminated germ-cell tumors (GCTs) failing chemotherapy and with adverse prognostic factors: The TAXIF II protocol. *Journal of Clinical Oncology.* 2009;27(15_suppl):5028-5028.

143. JPRN-UMIN000002398. Phase II study of intensive chemotherapy in treating pediatric patients with newly diagnosed cranial nongerminomaous germ cell tumor. In. Japanese Pediatric Brain Tumor Consortiun N, trans2009.

144. Center MDAC. High-dose Chemotherapy for Poor-Prognosis Relapsed Germ-Cell Tumors. In: <https://ClinicalTrials.gov/show/NCT00936936>; 2009.

145. Lorch A, Neubauer A, Hackenthal M, et al. High-dose chemotherapy (HDCT) as second-salvage treatment in patients with multiple relapsed or refractory germ-cell tumors. *Annals of Oncology.* 2010;21(4):820-825.

146. Feldman DR, Sheinfeld J, Bajorin DF, et al. TI-CE high-dose chemotherapy for patients with previously treated germ cell tumors: results and prognostic factor analysis. *J Clin Oncol.* 2010;28(10):1706-1713.

147. Lorch A, Rick O, Wündisch T, Hartmann JT, Bokemeyer C, Beyer J. High dose chemotherapy as salvage treatment for unresectable late relapse germ cell tumors. *J Urol.* 2010;184(1):168-173.

148. Fondazione Irccs Istituto Nazionale dei Tumori M, National Cancer I. High-Dose Chemotherapy and Stem Cell Transplant in Treating Patients With Metastatic Germ Cell Tumors That Have Not Responded to First-Line Therapy. In: <https://ClinicalTrials.gov/show/NCT01172912>; 2010.

149. Daugaard G, Skoneczna I, Aass N, et al. A randomized phase III study comparing standard dose BEP with sequential high-dose cisplatin, etoposide, and ifosfamide (VIP) plus stem-cell support in males with poor-prognosis germ-cell cancer. An intergroup study of EORTC, GTCSG, and Grupo Germinal (EO. *Ann Oncol.* 2011;22(5):1054-1061.

150. Olofsson SE, Tandstad T, Jerkeman M, et al. Population-based study of treatment guided by tumor marker decline in patients with metastatic nonseminomatous germ cell tumor: a report from the Swedish-Norwegian Testicular Cancer Group. *J Clin Oncol.* 2011;29(15):2032-2039.

151. Asirwa FC, Einhorn LH. Salvage therapy with high-dose chemotherapy (HDCT) and peripheral blood stem cell transplant (PBSCT) in patients with primary mediastinal nonseminomatous germ cell tumors (PMNSGCT). *Journal of Clinical Oncology.* 2011;29(15_suppl):e15119-e15119.

152. Haugnes HS, Laurell A, Stierner U, et al. High-dose chemotherapy with autologous stem cell support in patients with metastatic non-seminomatous testicular cancer - a report from the Swedish Norwegian Testicular Cancer Group (SWENOTECA). *Acta Oncol.* 2012;51(2):168-176.

153. De Giorgi U, Rosti G, Kopf B, et al. Multi-Cycle High-Dose Chemotherapy with TI-CE Regimen for Patients with Relapsed/Refractory Germ Cell Tumors &#x2013; a Single Institution Experience. *Annals of Oncology.* 2012;23:ix285-ix286.

154. Hartmann JT, Metzner B, Binder C, et al. Addition of Darbepoetin Alfa to Sequential High Dose Vip Chemotherapy for Patients with Advanced Metastatic Germ Cell Cancer. *Annals of Oncology.* 2012;23:ix284.

155. Courtney C, Virginia S, Betty RF, Paul Henry F, Kim Allyson M, Sumanta Kumar P. Paclitaxel-based high-dose chemotherapy (HDCT) for relapsed or refractory germ cell tumors (GCTs): Clinical outcome and quality of life (QOL) in long-term survivors. *Journal of Clinical Oncology.* 2012;30(15_suppl):e16563-e16563.

156. Nct, Masonic Cancer Center UoMN. Auto Transplant for High Risk or Relapsed Solid or CNS Tumors. In:2012.

157. Suleiman Y, Siddiqui BK, Brames MJ, Abonour R, Einhorn LH. Salvage Therapy with High-Dose Chemotherapy and Peripheral Blood Stem Cell Transplant in Patients with Primary Mediastinal Nonseminomatous Germ Cell Tumors. *Biology of Blood and Marrow Transplantation.* 2013;19(1):161-163.

158. Nct, Assistance Publique - Hôpitaux de Paris N. Salvage Chemotherapy for Poor Prognosis Germ Cell Tumors. In:2013.

159. Selle F, Wittnebel S, Biron P, et al. A phase II trial of high-dose chemotherapy (HDCT) supported by hematopoietic stem-cell transplantation (HSCT) in germ-cell tumors (GCTs) patients failing cisplatin-based chemotherapy: the Multicentric TAXIF II study. *Ann Oncol.* 2014;25(9):1775-1782.

160. Robertson PL, Jakacki R, Hukin J, Siffert J, Allen JC. Multimodality therapy for CNS mixed malignant germ cell tumors (MMGCT): results of a phase II multi-institutional study. *J Neurooncol.* 2014;118(1):93-100.

161. Andrea N, Manuela B, Rosalba M, et al. Analysis of the contemporary use of high-dose chemotherapy (HDCT) in germ cell tumors (GCT) in Europe: Early findings of an ongoing EBMT-sponsored study. *Journal of Clinical Oncology.* 2014;32(15_suppl):e15536-e15536.

162. Yago N, Shi-Ming T, Roy BJ, et al. Phase 2 trial of bevacizumab (BEV)/high-dose chemotherapy (HDC) with autologous stem-cell transplant (ASCT) for refractory germ-cell tumors (GCT). *Journal of Clinical Oncology.* 2014;32(15_suppl):4517-4517.

163. Andrea N, Rosalba M, Marco B, et al. Impact of response to induction chemotherapy (CT) and prior paclitaxel (TXL)-based CT on the outcome of salvage high-dose chemotherapy (HDCT) for relapsed germ-cell tumors (GCT) in the modern era: An EBMT Solid Tumors Working Party study. *Journal of Clinical Oncology.* 2015;33(15_suppl):4535-4535.

164. Nieto Y, Tu SM, Bassett R, et al. Bevacizumab/high-dose chemotherapy with autologous stem-cell transplant for poor-risk relapsed or refractory germ-cell tumors. *Annals of Oncology.* 2015;26(10):2125-2132.

165. Feldman DR, Glezerman I, Patil S, et al. Phase I/II Trial of Paclitaxel With Ifosfamide Followed by High-Dose Paclitaxel, Ifosfamide, and Carboplatin (TI-TIC) With Autologous Stem Cell Reinfusion for Salvage Treatment of Germ Cell Tumors. *Clin Genitourin Cancer.* 2015;13(5):453-460.

166. Goldman S, Bouffet E, Fisher PG, et al. Phase II Trial Assessing the Ability of Neoadjuvant Chemotherapy With or Without Second-Look Surgery to Eliminate Measurable Disease for Nongerminomatous Germ Cell Tumors: A Children's Oncology Group Study. *J Clin Oncol.* 2015;33(22):2464-2471.

167. Necchi A, Mariani L, Di Nicola M, et al. High-dose sequential chemotherapy (HDS) versus PEB chemotherapy as first-line treatment of patients with poor prognosis germ-cell tumors: mature results of an Italian randomized phase II study. *Ann Oncol.* 2015;26(1):167-172.

168. DeFilipp Z, Rosand CB, Goldstein DA, et al. High-Dose Chemotherapy and Autologous Stem Cell Transplantation for Previously Treated Germ Cell Tumors: A Single-Center Experience. *Biology of Blood and Marrow Transplantation.* 2016;22(3):S421-S422.

169. Egan G, Cervone KA, Philips PC, Belasco JB, Finlay JL, Gardner SL. Phase I study of temozolomide in combination with thiotepa and carboplatin with autologous hematopoietic cell rescue in patients with malignant brain tumors with minimal residual disease. *Bone Marrow Transplant.* 2016;51(4):542-545.

170. Necchi A, Miceli R, Bregni M, et al. Prognostic impact of progression to induction chemotherapy and prior paclitaxel therapy in patients with germ cell tumors receiving salvage high-dose chemotherapy in the last 10 years: a study of the European Society for Blood and Marrow Transplantation Solid Tumors Working Party. *Bone Marrow Transplantation.* 2016;51(3):384-390.

171. Drks, European Organisation for R, Treatment of Cancer EN. A Randomized Phase III Study of Sequential High-Dose Cisplatinum/Etoposide/Ifosfamide Plus Stem Cell Support Versus BEP in Patients With Poor Prognosis Germ Cell Cancer. In:2016.

172. Nct, Samsung Medical Center N. Reduced Dose Radiotherapy Following High Dose Chemotherapy in Intracranial Non-germinomatous Germ Cell Tumor. In:2016.

173. Moeung S, Chevreau C, Broutin S, et al. Therapeutic Drug Monitoring of Carboplatin in High-Dose Protocol (TI-CE) for Advanced Germ Cell Tumors: Pharmacokinetic Results of a Phase II Multicenter Study. *Clin Cancer Res.* 2017;23(23):7171-7179.

174. Maitri K, Nabil A, John M, Rafat A, Nasser HH, Lawrence HE. High-dose chemotherapy (HDCT) plus peripheral-blood stem-cell transplant (PBSCT) for patients (pts) with relapsed germ-cell tumors (GCT) and active brain metastases (mets). *Journal of Clinical Oncology.* 2017;35(15_suppl):4558-4558.

175. Thomas K, Franziska C, Fabian Maximilian M, et al. Phase II-study of sequential high-dose-chemotherapy with paclitaxel, ifosfamide, carboplatin, etoposide( P-ICE) in patients with relapsed or refractory germ cell tumors (GCT). *Journal of Clinical Oncology.* 2017;35(15_suppl):4552-4552.

176. Yago N, Shi-Ming T, Matthew TC, et al. Infusional gemcitabine + docetaxel/melphalan/carboplatin (GemDMC) ± bevacizumab (BEV) as an effective high-dose chemotherapy (HDC) regimen for refractory of poor-risk relapsed germ-cell tumors (GCT). *Journal of Clinical Oncology.* 2017;35(15_suppl):4519-4519.

177. Gössi F, Spahn M, Samaras P, Beyer J, Schardt J, Pabst T. Response to first-line treatment and histology are associated with achieving complete remission after the first salvage high-dose chemotherapy in relapsing germ cell tumor patients. *Bone Marrow Transplantation.* 2018;53(7):820-825.

178. Deepak K, Parameswaran H, Muna Q, et al. Tandem high-dose chemotherapy and autologous hematopoietic stem cell transplantation (SCT) compared to single SCT for relapsed/refractory germ cell tumors (GCT). *Journal of Clinical Oncology.* 2018;36(6_suppl):572-572.

179. Osorio DS, Dunkel IJ, Cervone KA, et al. Tandem thiotepa with autologous hematopoietic cell rescue in patients with recurrent, refractory, or poor prognosis solid tumor malignancies. *Pediatr Blood Cancer.* 2018;65(1).

180. Nabil A, Costantine A, Rafat A, et al. Survival and toxicity outcomes in patients age 40 or older with relapsed metastatic germ cell tumors (mGCT) treated with high-dose chemotherapy (HDCT) and autologous peripheral-blood stem cell transplant (PBSCT). *Journal of Clinical Oncology.* 2019;37(7_suppl):522-522.

181. Callera AF, Rosa ES, Callera F. Intermediate-dose cytarabine plus G-CSF as mobilization regimen for newly diagnosed multiple myeloma and heavily pre-treated patients with hematological and non-hematological malignancies. *Transfusion and Apheresis Science.* 2019;58(3):318-322.

182. Bilal A, Reem A, Sandra KA, et al. Maintenance oral etoposide (VP-16) after high-dose chemotherapy (HDCT) for patients with relapsed metastatic germ-cell tumors (mGCT). *Journal of Clinical Oncology.* 2020;38(15_suppl):5051-5051.

183. Nabil A, Sandra KA, Rafat A, et al. Prognostic significance of rate of tumor marker (TM) decline during high-dose chemotherapy (HDCT) for relapsed germ cell tumors (rGCT). *Journal of Clinical Oncology.* 2020;38(6_suppl):403-403.

184. Vaibhav A, Sandra KA, Rafat A, et al. High-dose chemotherapy (HDCT) and peripheral-blood stem cell transplant (PBSCT) in patients age 40 or older with relapsed metastatic germ-cell tumors (mGCT). *Journal of Clinical Oncology.* 2020;38(15_suppl):e17054-e17054.

185. Nct, Yes MDACC. Chemotherapy and Donor Stem Transplant for the Treatment of Patients With High Grade Brain Cancer. In:2020.

186. Nct, Sun Yat-sen University Y. Nab-PTX, Ifosfamide and Cisplatin in the Treatment of Pediatric Extracranial Germ Cell Tumor. In:2020.

187. Chevreau C, Massard C, Flechon A, et al. Multicentric phase II trial of TI-CE high-dose chemotherapy with therapeutic drug monitoring of carboplatin in patients with relapsed advanced germ cell tumors. *Cancer Med.* 2021;10(7):2250-2258.

188. Nabil A, Indiana U. Maintenance Oral Etoposide or Observation Following High-dose Chemo for GCT. In: <https://ClinicalTrials.gov/show/NCT04804007>; 2021.

189. Madanchi R, Engel NW, Alsdorf W, et al. Approaches of stem cell mobilization in a large cohort of metastatic germ cell cancer patients. *Bone Marrow Transplantation.* 2022;57(5):729-733.

190. Fadi T, Rafat A, Sandra KA, et al. Salvage high-dose chemotherapy (HDCT) for relapsed primary mediastinal nonseminomatous germ-cell tumors (PMNSGCT). *Journal of Clinical Oncology.* 2022;40(16_suppl):5032-5032.

191. Ryan A, Nabil A, Rafat A, et al. Randomized phase 2 trial of maintenance oral etoposide or observation following high-dose chemotherapy for relapsed metastatic germ cell tumor. *Journal of Clinical Oncology.* 2022;40(6_suppl):TPS429-TPS429.

192. Nct, Yonsei University N. Paclitaxel/Ifosfamide/Cisplatin Chemotherapy for High Risk Pediatric Germ Cell Tumor. In:2022.

193. Taza F, Abonour R, Zaid MA, et al. Maintenance Oral Etoposide After High-Dose Chemotherapy (HDCT) for Patients With Relapsed Metastatic Germ-Cell Tumors (mGCT). *Clin Genitourin Cancer.* 2023;21(2):213-220.

194. 44th Congress of the International Society of Paediatric Oncology (SIOP) 2012, London, United Kingdom, 5th–8th October, 2012 SIOP abstracts. *Pediatric Blood & Cancer.* 2012;59(6):965-1152.
